# Low-cost assembly of a cacao crop genome is able to resolve complex heterozygous bubbles

**DOI:** 10.1038/s41438-019-0125-7

**Published:** 2019-04-06

**Authors:** Joe Morrissey, J. Conrad Stack, Rebecca Valls, Juan Carlos Motamayor

**Affiliations:** Mars Chocolate, 13601 Old Cutler Road, Miami, FL 33158 USA

**Keywords:** Genetics, Genomics

## Abstract

Cacao (*Theobroma cacao*) is a tropical tree that produces the essential raw material for chocolate. Because yields have been stagnant, land use has expanded to provide for increasing chocolate demand. Assembled genomes of key parents could modernize breeding programs in the remote and under-resourced locations where cacao is grown. The MinION, a long read sequencer that runs off of a laptop computer, has the potential to facilitate the assembly of the complex genomes of high-yielding F_1_ hybrids. Here, we validate the MinION’s application to heterozygous crops by creating a de novo genome assembly of a key parent in breeding programs, the clone Pound 7. Our MinION-only assembly was 20% larger than the latest released cacao genome, with 10-fold greater contiguity, and the resolution of complex heterozygosity and repetitive elements. Polishing with Illumina short reads brought the predicted completeness of our assembly to similar levels to the previously released cacao genome assemblies. In contrast to previous cacao genome projects, our assembly required only a small scientific team and limited reagents. Our sequencing and assembly methods could easily be adopted by under-resourced breeding programs, speeding crop improvement in the developing world.

## A sweet solution for studying cacao genetics

Genomic insights from an efficient, low-cost DNA sequencing strategy could enable more sustainable cacao cultivation. Growing demand for chocolate is currently being satisfied by increasing land use. A richer understanding of this plant’s genome could enable development of more productive strains, and Juan Carlos Motamayor and colleagues at Mars Chocolate have demonstrated a method for rapidly collecting such data. Most sequencing platforms produce vast multitudes of short DNA sequences which must then be assembled into a final map—a particular challenge for cacao, whose genome contains complex repetitive regions that are tough to reconstruct. Using a newer platform known as MinION, which produces far longer sequence reads, Motamayor’s team assembled a detailed genome map for a widely-used cacao cultivar within months for under $5,000. This approach could facilitate genetic engineering efforts to improve crop performance.

## Introduction

**Fig. 1:**
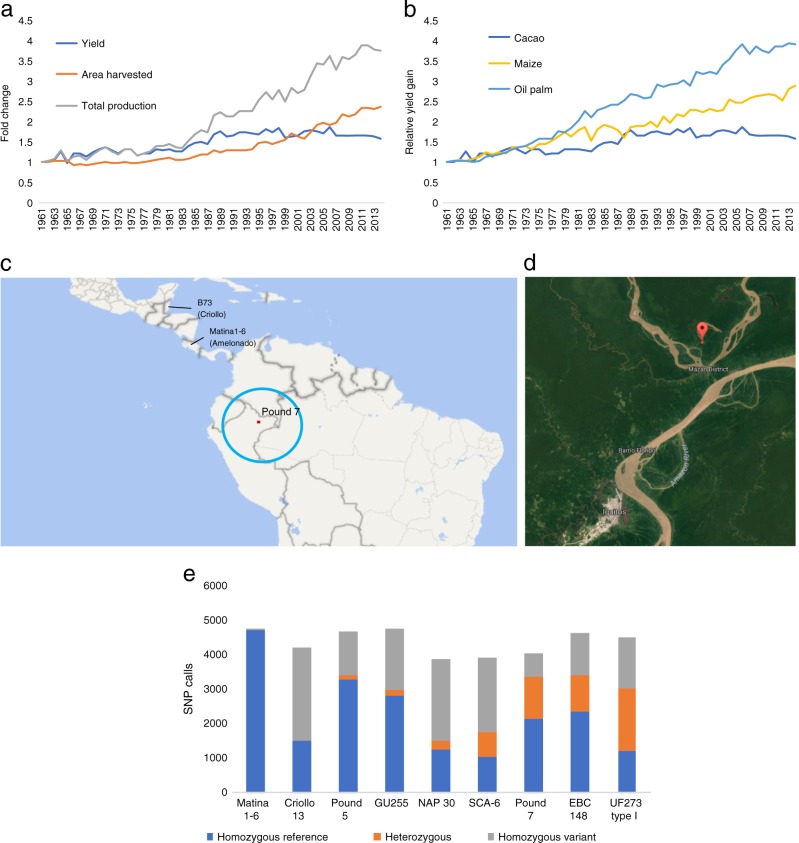
Pound 7 is better representative of both wild cacao and elite F_1_ hybrids than the previous cacao reference genomes. **a** Relative change in worldwide cacao yields, compared with the change in total production and the amount of land used to produce the cacao, normalized to the 1961 data points. Data from FAO.org. **b** Relative worldwide yield gains for cacao compared with the two other crops. Data from FAO.org. Values are relative, normalized to the 1961 datapoint. **c** The site of collection of Pound 7 is represented by the red square. The center of cacao’s diversity is the blue circle^13,14^. The previous cacao reference genomes are Matina 1–6 (associated with Costa Rica)^5^ and B97–61/B2 (associated with Belize)^3,4^. The satellite image (adapted from Google Maps) shows the coordinates of where Pound 7 was collected, 73.10 W 3.45 S (Pound, 1943; Turnbull, C.J. and Hadley, International Cocoa Germplasm Database). **d** Pound 7 is a heterozygous wild hybrid of several ancestral groups (Motamayor et al.^12^), in contrast to the two domesticated cacao that were used for the previous reference genomes. **e** The number of heterozygous SNP calls out of 135,696 SNPs from resequenced cacao genomes^41^ shows the range of heterozygosity in cacao accessions. The current reference genomes are the “highly homozygous” B97–61/B2^3,4^ and Matina 1–6^5^. Both are relatively homozygous compared with Pound 7 and the widely cultivated F_1_ hybrid CCN 51

Cacao is a diploid tree that produces the raw material for the chocolate industry, and is grown primarily in the tropics of West Africa, Southeast Asia, and South and Central America. While recent production levels have increased, this has come from expansion of the land devoted to cacao. Yield, in contrast, has stagnated (Fig. 1a, b). The killing of fragile tropical ecosystems to expanded cacao production could be prevented by modernizing under-resourced breeding programs in the developing world using genomics technologies, improving yields on existing farms.

Genomic approaches are now readily utilized to advance breeding programs in staple crops^1^, and can even impact the production of tropical commodities like oil palm^2^. While the cost of second-generation sequencing has rapidly decreased, the reads produced (e.g., short, paired, low-error rate) are not well-suited to directly resolve repetitive elements and structural heterozygosities of complex crop genomes. The first two sequenced cacao genomes, B97–61/B2^3,4^ (“Criollo”) and Matina 1–6^5^, were selected for their relative homozygosity. Neither of them are representative of elite cacao varieties, which are heterozygous F_1_ hybrids that are propagated through grafting. Expanding the current set of genomic resources to include the heterozygous genetic structures of high-yielding F_1_ hybrids is an important tool for increasing yields without further expanding land use.

The MinION is a palm-sized sequencer that runs off of a laptop computer, and that which is capable of producing long reads ranging from tens to kilobases in length^6^. Reads in this size range have the potential to accurately resolve heterozygous haplotypes. Moreover, the small size of the device allows it to be used in underdeveloped or remote locations, as demonstrated during the Ebola outbreak in West Africa^7^. Cacao is the ideal model to evaluate MinION sequencing of complex crop genomes, as two high-quality reference genomes exist, one of which was recently improved by the addition of PacBio long reads^4^.

Here, we validate the MinION by sequencing the cacao clone Pound 7, a wild tree collected by F.J. Pound from a riverbank in Peru in 1942 (Fig. 1c, d^8^). Pound 7 is held in many germplasm collections and has been widely used in cacao breeding programs (Supplementary Table 1). It served as a cornerstone of the Peruvian and Costa Rican breeding programs due to its high-yielding progeny^9,10^ and resistance to black pod (*Phytophthora spp*.)^11^.

Cacao is preferentially outcrossing, and consequently wild or primal plants often have a higher level of heterozygosity, as seen in Pound 7 (Fig. 1e), an admixture of four primary ancestral genetic groups^11^. In contrast, Matina 1–6 is a pure Amelonado, and B97–61/B2 is a pure Criollo;^12^ both are domesticated, and inbreeding has resulted in relatively homozygous genomes^3–5^. The genetic distance between Pound 7 and these clones is relatively high^11–14^ (Supplementary Table 2), and Pound 7 is much more heterozygous, making it more structurally representative of a cultivated F_1_ hybrid (Fig. 1e).

We present a highly contiguous, and high-quality genome assembly for the Pound 7 cacao clone. We validated the assembly against two BAC-derived haplotype assemblies from a single locus in the Pound 7 genome, where extreme structural differences are present between the two haplotypes. While the previous two cacao genome projects took years and required large investments of money and labor, by leveraging the MinION platform, we have rapidly produced a similar quality assembly of a much more complex, heterozygous genome for a relatively low cost in materials (< $5,000 at the time of writing), with tools that can function in the under-resourced locations where cacao is produced.

## Results

### Sequencing of Pound 7 with MinION

To better test if the MinION would function in remote cacao breeding sites, the DNA preparation was simplified as much as possible. High-molecular-weight DNA from the “Pound 7” tree held at the Mars greenhouse in Miami, USA, was prepared, and unfragmented DNA (with no size selection) was used in with a standard “1D” library kit for four flow cells. One additional flow cell, a 1D library, used low-molecular-weight DNA (12-kbps fragments). The output ranged from ~2 to 6 gbps per flow cell (Table 1). The highest total output was achieved with the 12-kbps fragment library, although the N50 of the reads was much lower. The majority of individual runs with non-fragmented DNA had read N50s greater than 20,000 bps, with the lowest N50 of any flow cell above 10 kbps.

**Table 1:** Statistics for MinION sequencing runs

Number	Flowcell	gbps	No. of reads	Read N50 (bp)	Read L50
1	FLO-MIN106	3.21	294,365	13,771	85,335
2	FLO-MIN107	4.89	381,720	22,023	71,713
3	FLO-MIN106	5.82	1,952,520	5705	301,955
4	FLO-MIN106	2.99	287,799	20,389	47,243
5	FLO-MIN106	2.25	197,470	22,591	32,643

The “gbps” column represents the sum total of nucleotides produced by each flowcell, using only sequences that passed Albacore’s low-quality filter. All runs used the 1D (SQK-LSK108) library preparation kit. Flow cell number three used the 12 -kbps fragment enriched library. Flow cell number four was stored at room temperature for several days while in possession of US Customs.

### De novo assembly of the MinION-only Pound 7 genome

A total of 18.6 gbps of MinION reads filtered for quality using the Albacore defaults were used in the genome assembly. This represents approximately 42X coverage of the Pound 7 haploid genome, which was previously estimated at 442 mbps by flow cytometry^5^. For comparison, the genome size of Matina 1–6 was estimated to be 445 mbps^5^, and B97–61/B2 has been estimated at both 409 and 430 mbps^3,5^.

The MinION reads were assembled twice using different strategies that have been suggested in recent literature: one strategy was based on minimap and miniasm^15^ (“minimap”) and the other on canu and SMARTdenovo^16^ (“canusm”). The contigs from both the minimap and canusm assemblies were polished, split at sites where there was evidence of a misjoin, and scaffolded using publicly available, SNP-based linkage maps, including a map specific to Pound 7. Contig statistics at each major step of assembly and scaffolding are presented in Table 2.

**Table 2:** Comparison of cacao genome assemblies

	Cultivar	Criollo V2	Matina 1–6	Pound7											
	Assembly data/method	PacBio RSI, 454, Illumina, Sanger of BACs	454, Illumina, Sanger of BACs	minimap–miniasm–racon						Canu-SMARTde novo					
	Version/stage	v2.0	v1.1	Unpolished	Polished	Split (by coverage)	Nonredundant	Split (by map consensus)	Final draft	Unpolished	Polished	Split (by coverage)	Nonredundant	Split (by map consensus)	Final draft
Contiguity	Total length (bp)	306,380,296	330,821,837	392,822,621	393,460,530	388,496,692	379,301,597	379,301,597	390,893,205	341,510,689	345,320,506	345,043,684	344,633,077	344,633,077	345,527,408
	N. contigs	7743	20,103	1038	1038	1834	1506	1517	2720	663	663	776	730	769	813
	Longest contig (bp)	936,847	1,030,196	4,922,249	4,933,427	4,932,277	4,932,277	4,932,277	4,932,277	9,646,223	9,762,425	8,557,809	8,557,809	8,557,809	9,762,425
	Shortest contig (bp)	1004	150	2223	2220	1001	1001	619	619	9898	10,028	1009	1032	627	627
	Median contig length (bp)	17,900	1,369	151,556	151,878	65,356	88,375	88,602	5518	166,639	166,970	134,792	155,726	146,729	107,747
	N50 (bp)	87,439	84,396	906,913	907,755	769,569	803,644	781,587	757,789	1,402,148	1,425,322	1,284,321	1,284,321	1,256,900	1,326,996
	N90 (bp)	18,795	14,971	140,537	140,664	127,828	142,289	142,272	122,633	276,232	280,231	262,563	265,332	250,503	253,804
	N95 (bp)	11,288	5318	97,563	97,802	81,742	94,211	94,211	76,298	130,841	132,654	126,970	131,288	118,665	118,665
	L50	879	1080	124	124	142	136	138	145	66	66	74	74	78	70
	L90	3776	4380	550	551	627	570	576	651	282	282	300	299	313	299
	L95	4810	6190	719	719	816	732	738	851	374	374	396	393	413	398
	% GC	34.09%	34.01%	34.03%	33.84%	33.86%	33.87%	33.87%	33.85%	33.79%	33.96%	33.96%	33.96%	33.96%	33.96%
BUSCO v3	Complete, total (%)	95.14%	95.83%	62.57%	95.49%				95.21%	54.38%	95.35%				95.28%
	Complete, total (N)	1370	1380	901	1375				1371	783	1373				1372
	Complete, single copy	1350	1356	875	1335				1325	772	1346				1342
	Complete, duplicated	20	24	26	40				46	11	27				30
	Fragmented	24	22	103	22				21	112	23				23
	Missing	46	38	436	43				48	545	44				45

Table of contig statistics for both reference genomes, and for each stage of (both) assemblies of Pound 7. For each Pound 7 assembly, the stages are listed in the left-to-right order starting with the “uncorrected” assembly and ending with the “final draft” set of contigs. Splitting steps were checked to make sure that they did not partially intersect BUSCO hits. Note: BUSCO summaries for both “final draft” assemblies were run using v3.0.2, on a different computer.

The minimap and canusm assembly methods produced a comparable number of contigs; however, the minimap assembly was substantially larger (> 45 Mbps) and less contiguous (~40% lower N50). Some of this difference can be attributed to the higher degree of redundancy in the minimap assembly. Redundant contigs are defined as the shorter of two contigs sharing 70% common sequence at 90% or above sequence identity. This redundancy can be attributed to heterozygosity or structural variation in the genome, or to the high-error rate of the assembled reads, which could result in uncollapsed haplotypes. In the minimap assembly, 12 Mbps of redundant contigs were identified, while the total redundancy present in the canusm assembly was less than 1 Mbps (Supplementary Table 3).

Both Pound 7 assemblies were larger and more contiguous than the current available reference genomes (Table 2). When compared with the PacBio-improved B97–61/B2 v2 assembly published in 2017^3,4^, the minimap assembly was over 20% larger, and had seven times fewer contigs (1038 vs. 7743). The canusm assembly was ~10% larger, and had approximately 10 times fewer contigs (813 vs. 7743). The contig N50 values of minimap and canusm assemblies were an order of magnitude greater than the current reference genomes (Table 2). The larger size of the Pound 7 assemblies compared with the two reference genomes can be partially attributed to redundancy of contigs. This is consistent with the higher level of heterozygosity of Pound 7 vs. both B97–61/B2 and Matina 1–6 (25% of loci are heterozygous in Pound 7, compared with 1–2% for B97–61/B2 and Matina 1–6, Fig. 1e).

### Assembly scaffolding

A genetic linkage map specific to Pound 7 was created using SNP genotyping data from two F_1_ mapping populations, where Pound 7 was the mother^5^. In an earlier study, 5218 genotypes were called for 250 progeny, Pound 7, and both male parents (cultivars UF-273 type 1 and type 2) using a custom Illumina Infinium beadtype array^17^. A total of 1065 loci were found to be informative about maternal linkage, and 1034 of these were uniquely placed and ordered in 10 linkage groups using LepMap2^18^. This map was largely consistent with a previously developed map from the same populations that used the cross-pollinator (CP) approach (Supplementary Fig. 2)^5^.

After mapping the Illumina SNP chip sequences to each set of “final draft” Pound 7 contigs (Table 2), we found that 99.8% of SNP alleles from the minimap contigs and 99.9% of SNP alleles from the canusm contigs matched one of the corresponding Pound 7 alleles from the Illumina chip (Supplementary Table 5). No inconsistencies were observed between the Pound 7 linkage map and the minimap assembly, while four contigs in canusm were inconsistent (Supplementary Table 4, Supplementary Figure 5). A total of 178,509,798 bp (47.1%) from the minimap assembly and 210,588,373 bp (61.1%) from the canusm assembly were anchored to the Pound 7 linkage map by ALLMAPS^19^ (Table 2).

The Pound 7 contigs were scaffolded using the Pound 7 linkage map and five additional publicly available SNP linkage maps from earlier studies^4,17,20^. All five of these additional maps were derived from F_1_ mapping populations, in which Pound 7 was not directly represented. The most dense of these additional maps contained approximately four times the number of placed SNPs compared with the Pound 7 map (Supplementary Table 4). While scaffolding with non-Pound 7 maps produces a chromosome-level genome, which may not conserve structural variation specific to Pound 7, it does allow many more contigs to be anchored into pseudomolecules (i.e., chromosomes). The results from scaffolding with the Pound 7 map alone and all six linkage maps are shown in Table 4. For the minimap assembly, a total of 327 mbps of contigs (86% of the total) were placed onto the 10 linkage groups of cacao. Three contigs minimap assembly were flagged as having potential misjoins (i.e., where discrepancies were observed in four or more out of the six linkage maps). For the canusm assembly, 317 mbps of contigs (92% of the total) were placed, with nine contigs flagged as having potential misjoins. The higher contiguity of the canusm assembly can explain the higher percentage of anchored bases, and the larger number of identified misjoins, compared with the minimap assembly. Although the percent anchored is lower in the Pound 7 assemblies compared with the previously released reference genomes, the total number of bases anchored in the former are comparable with the latter (Table 4).

### Annotation, validation, and comparison

The Pound 7 contigs were evaluated with BUSCO v3^21^ at different stages of the assembly process. For both the minimap and canusm assemblies, the BUSCO statistics were greatly improved after polishing with Pilon^22^ using a moderate amount of low-error rate Illumina paired-end reads (~30× coverage of 100 × 2 -bp read pairs). The polishing resulted in a negligible change in the assembly sizes and no change to the number of contigs, but improved the BUSCO score from 63% to 95% for the minimap assembly and from 54% to 95% for the canusm assembly (Table 2). Interestingly, the profile of corrections made by Pilon were fairly different between the two assembly methods (Supplementary Table 6). After the first iteration of Pilon, when most corrections were made, the number of insertions, deletions, and substitutions were tallied. For minimap, the number of insertion, deletion, and substitution corrections are roughly similar. For canusm, insertions accounted for nearly 80% of corrections. Whatever the reason for this bias, the end result for both methods seemed to be a substantial improvement in sequence-level quality of the contigs.

The BUSCO completeness scores for the minimap and canusm assemblies were both greater than 95%, showing a similar level of completeness as the two reference genomes (Table 2). There were some differences in the BUSCO gene models found within the two Pound 7 assemblies and both reference genomes (Supplementary Table 7; Supplementary Figure 6). Most of them are minor (e.g., “complete” in one assembly, but “fragmented” in another), and probably reflect a lack of robustness in the BUSCO assessment process. Indeed, at least one instance when assessing the canusm assembly, a “Complete” single-copy BUSCO hit from an earlier stage of the assembly was “Missing” in a later stage, despite no changes to the contig where it was originally found. However, there were some noteworthy differences among the BUSCO gene models found in the four assemblies. The minimap contigs were found to have the highest incidence of duplicated BUSCO gene models (Supplementary Table 7). While this is consistent with the higher degree of redundancy present in that assembly, the copy numbers of several BUSCO genes were substantially higher in the minimap versus other assemblies. The BUSCO gene EOG093603JD was found eight times in minimap, three times in canusm, and five times in each reference genome. In the reference genomes, all five copies were spread among five different chromosomes. In the minimap assembly, five copies were placed on different chromosomes, but three additional instances were found in tandem with one of the hits. This tandem duplication does not exist in the canusm assembly. An investigation of remapped long (> 20 kbp) MinION reads indicated that the region containing the tandem duplications was covered reasonably well (~20 ×), including some MinION reads that spanned the entire region without break. Furthermore, a comparison between this region from the minimap and canusm assemblies showed very little structural difference (Supplementary Figure 7A) and high-nucleotide identity (> 99%); a comparison with the matina and criollo reference showed the same (Supplementary Figure 7B). These observations support the idea that tandem replication is real, but simply was not picked up by BUSCO in the canusm assembly for some reason (e.g., poor sequence-level polishing). The other notable exception was for the BUSCO gene EOG09360D08, where the difference in the number found in each assembly was more extreme. The gene was found three times, in tandem, in both reference genomes, and four times, in tandem, in the canusm assembly. By contrast, the gene was found 19 times in the minimap assembly, replicated in tandem on three different contigs that were not identified as separate haplotypes from the same region. As before, the three regions of the minimap assembly where hits were located, can be found in the canusm assembly, with little-to-no structural differences between them. These regions also appear to be intact in both the criollo and matina genomes, although large gaps in these regions of the reference genomes make a 1-to-1 comparison difficult. Regardless, the regions in minimap contigs where EOG09360D08 was found are highly repetitive, and it is possible that the number of minimap hits was higher compared with the other assemblies in part due to the way BUSCO ran (e.g., differences in parameter auto-tuning), or differences in polishing, rather than structural errors in the minimap assembly. Care should be taken to not over-interpret BUSCO scores, but it was useful to investigate discrepancies found between different assembly methods.

### Ab initio gene models

While complete gene model prediction is outside the scope of this paper, and we did not predict evidence-based gene models, ab initio predictions were made for each set of contigs using Augustus^23^ (Supplementary Table 8). These predicted gene models were assessed with BUSCO (in transcriptome mode) to further validate the sequence-level accuracy of the two Pound 7 genome assemblies. Nearly 88% of BUSCO transcripts were identified in the predicted (“ab initio”) gene models from the minimap and canusm. The same ab initio prediction was run on the two cacao reference genomes;^4,5^ the BUSCO completeness score for the two references ab initio run output was only around 1% higher to the Pound 7 scores (Supplementary Table 8). However, when BUSCO was run on the final refined coding domains published with each reference genome, the scores were higher (> 98%). The results from the ab initio comparison (88% vs. 89%), suggest that the genic regions in both Pound 7 assemblies are largely free of uncorrected InDels that would lead to fragmented transcript predictions and a lower BUSCO score.

### Repeat content and transposable elements

**Fig. 2:**
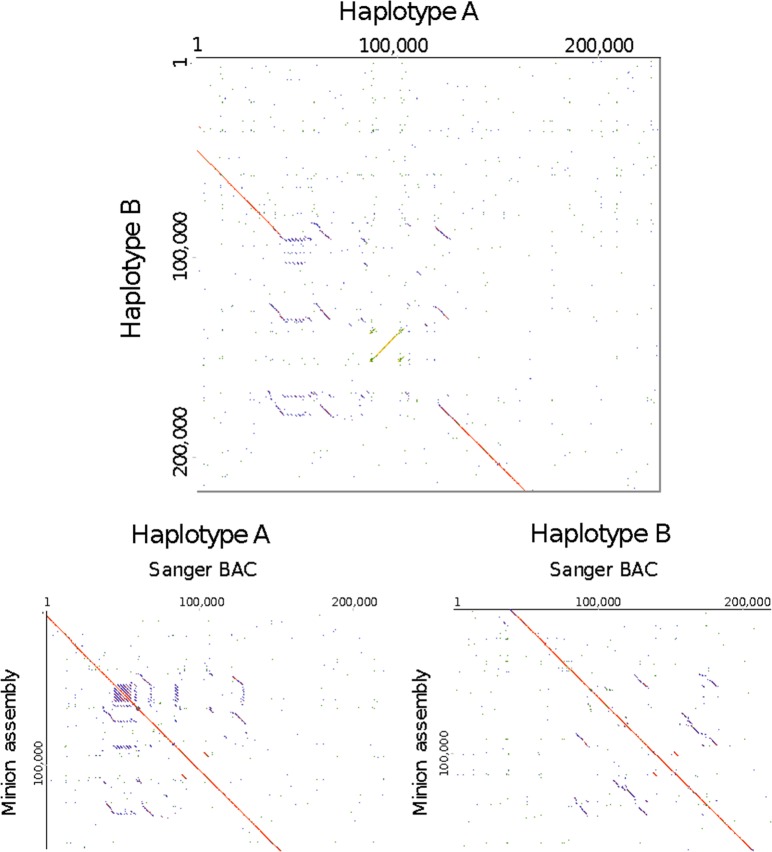
Our assembly resolved a highly heterozygous locus. The top panel is the alignment of BAC sequences for heterozygous haplotypes on Chromosome 4 of Pound 7. When aligned to the Matina 1–6 genome^5^, Haplotype A maps to Scaffold 4: 142672 to 324489, and Haplotype B maps to Scaffold 4: 181600 to 392073. The lower two panels show the two haplotypes resolved in the MinION assembly, aligned to the BAC sequences. A summary of the annotations is in Supplementary Table 10. Haplotype A is deposited on NCBI as P7SI_AltHap_V3 and Haplotype B is P7SI_MatHap_V3

Further analysis of the Pound 7 assemblies by repeat masking showed that their relatively large size compared with the reference genomes could be due in part to the resolution of additional repetitive sequences. The minimap assembly had nearly 50% more LTR retrotransposons assembled compared with Criollo B97–61/B2 v2, while the canusm had roughly 33% more (Table 3). The assembly of non-repetitive sequences was also higher than in the Matina 1–6 and B97–61/B2 v2 assemblies, respectively (Table 3). This is likely due to the heterozygous haplotypes of Pound 7 and the slightly larger genome size estimated by flow cytometry (Fig. 2, Supplementary Figure 3). Repetitive content was not overrepresented in the alternative haplotypes identified in either Pound 7 assembly when compared with the rest of the assembly (Supplementary Table 9). So, not all additional repetitive content could explain the redundancy found in the Pound 7 assemblies, although this redundancy was likely a conservative (under-)estimate.

**Table 3:** Comparison of retroelements in cacao genome assemblies

	B97–61/B2	Matina 1–6	Pound 7 minimap	Pound 7 canusm
Genome size by flow cytometry (mb)	430	445	442	442
Total assembly (mb)	306	331	393	345
Total repetitive (mb)	120.29 (39.26%)	136.60 (41.29%)	172.84 (43.93%)	148.31 (42.95%)
Retroelements (mb)	104.69 (34.17%)	120.59 (36.45%)	153.42 (38.99%)	131.65 (38.12%)
DNA transposons (mb)	10.07 (3.29%)	10.21 (3.09%)	12.21 (3.10%)	10.79 (3.13%)
Non-repetitive (mb)	186.09 (60.74%)	194.22 (58.71%)	220.62 (56.07%)	197.01 (57.05%)

Retroelements were identified with RepeatMasker using the Viridiplantae library26. RE stands for retroelement, and does not include all repetitive elements in the assembly. Percent values in the table represent percentage of the assembly. Flow-cytometry values are from refs. ^3,5^. For reference, the repetitive element content of the published cacao reference genomes has been analyzed previously, with varying results. The Criollo B97–61/B2 v1 repetitive element proportion of the genome was reported as 24%^3^ and 35%;^5^ the Criollo B97–61/B2 v2 at 15% by RepeatMasker and 32% by Windowmasker4; the Matina 1–6 V1.1 genome was reported as 42% repetitive elements^5^

### Long reads allow resolution of heterozygous loci

To determine if our assemblies could resolve the heterozygosity common in wild cacao and elite F_1_ hybrids, we examined a highly heterozygous, 150–200-kbps locus on chromosome 4 (Fig. 2). These complex haplotypes were previously only resolved with Sanger sequencing of BACs (NCBI Bioproject PRJNA421343). This locus contains several recently active transposable elements (TEs), multiple gene duplication events, and other structural variations (Fig. 2, top panel). We annotated 32 TEs and 20 non-TE gene models in Haplotype A, and 37 TEs and 28 non-TE gene models in Haplotype B (Supplementary Table 10).

In this region of the genome, the canusm assembly only contained one of the two haplotypes (Supplementary Figure X). The minimap assembly, in contrast, provided an accurate reconstruction of the structural heterozygosity (Fig. 2, bottom panels, Supplementary Figure 3), but the sequence-level identities were only 97.3% and 98.2% compared with our BAC-based assemblies. The low-sequence identity is likely due to the challenge of polishing complex repetitive elements like TEs with short reads. Indel differences between the minimap contigs and their BAC counterparts were observed 1.5 and 2.0 times more frequently than SNP differences, and were predominantly limited to homopolymer repeats. Both SNPs and indels were more frequent within the annotated TEs, especially within an ~17-kbps gypsy-like LTR element. This specific LTR is present in each haplotype, but it is inverted between the two; this complex structural variation was resolved in our assembly.

To determine if the relatively high-error rate in the region affects coding gene models, we aligned the two alleles of three separate genes from the BAC Sanger sequencing and our assembly (Matina 1–6 gene IDs Thecc1EG016795, Thecc1EG016799, Thecc1EG016803). The six alleles showed 100% identity between the BAC assemblies and our assembly and were in phase. This suggests that repetitive elements Minion-based assemblies will remain difficult to polish with short-insert paired-end reads, while protein-coding genes can be accurately resolved.

Additional bubbles of heterozygozity were identified computationally, resulting in 40 contig pairs (i.e., 80 of the 1038 contigs in the assembly) (Supplementary Table 11). When the first three pairs were compared manually, they showed alignment (Supplementary Figure 5).

## Discussion

### Positive aspects and limitations of our approach

Introgression of QTLs into elite lines has been a successful strategy in annual crops with advanced breeding programs, like rice and maize^24^. Numerous QTLs have been published for cacao^20,25–28^. But because of the long-generation times (2–4 years from seed to flowering), it could take decades to introgress a QTL into an elite cacao line, and remove the linkage drag from the QTL donor. Genome editing allows the direct improvement of elite cacao clones; however, CRISPR-based gene editing relies on highly specific gRNA constructs, requiring complete and accurate genomes assemblies of the lines being edited. This is critical since off-target effects were reported in early CRISPR experiments, and could become an obstacle to consumer acceptance of genome-edited food crops.

In the case of cacao, the original two cacao reference genomes were sequenced because of their homozygosity, and are likely of little value to CRISPR-based crop improvement due to their poor agronomic value. While generating and sequencing BACs can provide excellent haplotype resolution of complex regions, this process is labor-intensive. Both of our assemblies contained a mixture of haploid and diploid regions (Supplementary Figure 3), and one—minimap—was able to resolve the structural differences within a complex and a highly variable region of chromosome 4 (Fig. 2).

The first cacao genome published was the highly homozygous B97–61/B2 v1 genome, which was generated using 454 single reads, 454 paired-end reads, Illumina paired-end reads, and Sanger BAC end reads^3^. The second cacao genome published was the highly homozygous Matina 1–6 genome, which used a similar sequencing strategy^5^. The B97–61/B2 genome was recently improved (v2) with the incorporation of 52X coverage of PacBio long reads, and additional Illumina paired-end and mate-pair reads^4^ (Table 2).

The assemblies produced for this study were larger than the previously published cacao genomes. There are a few possible explanations for the 13–28% larger size of the Pound 7 genome assemblies compared with the current NCBI reference genome, B97–61/B2 (v2) (Table 1). To start, previous estimates of haploid genome size from flow cytometry indicate that the Pound 7 genome could be expected to be ~5–10% larger than B97–61^3,5^. Furthermore, the redundancy observed in each of the Pound 7 assemblies would clearly also account for some of the differences. Another plausible explanation is that the long MinION reads might have helped resolve more long (> 10 kbp) LTR retrotransposons, which are present in the cacao genome^42^.

The Pound 7 assemblies also had sevenfold fewer contigs, and a tenfold greater contig N50 compared with B97–61, while achieving an acceptable degree of accuracy once they had been polished. While the initial contigs produced using the high-error MinION reads alone seemed to contain many inaccuracies, they were an excellent starting point for subsequent refinement and polishing. Polishing was attempted with the MinION reads alone using the nanopolish software, but the runtime was gauged to be prohibitively long, even parallelizing across more than 32 CPUs of a modern, high-resource computer. Even had the runtime not been so high, it is possible that the obtained coverage of MinION reads (~40x of haploid genome) would not have been sufficient to accurately polish the Pound 7 assemblies. The use of low-error Illumina reads obtained at lower coverage were in contrast able to sharply improve BUSCO scores, bringing them to a similar level of completeness as the B97–61/B2 v2 and Matina 1–6 (v1.1) reference genomes (Table 2). For cacao and similarly sized genomes, the cost of paired, short-insert Illumina sequencing is likely less expensive than the computation time it would have taken to polish the assemblies using the MinION reads exclusively.

When comparing the whole-genome Pound 7 assemblies to haplotypes derived from BACs, the alignment differences fall mainly within annotated TEs and not genic regions. This likely reflects the fundamental difficulty of correcting long, repetitive regions in plant genomes using short-insert read pairs, an effect that is compounded when the TEs in the genome are active, and fewer genetic differences separate newly duplicated regions. Conversely, we expect that unique sequences within the genome, such as our annotated gene models and the gene models in the curated BUSCO set, will be easier to correct using the approach described. This haplotype comparison, the comparable number of gene models found ab initio, and the seeming quality of these gene models, suggest that MinION-based assemblies could greatly help resolve complex structural differences without a large negative impact on gene model prediction. Despite the problems presented by TEs, our results suggest that de novo resolution of complex haplotypes is possible with longer reads, which can be obtained more easily and cheaply than targeted BAC resequencing.

When Pound 7 contigs were anchored to publicly available linkage maps from other cacao clones, ~3% of the contigs were flagged as “false” joins. These could represent structural variations in Pound 7, flaws in our assembly, or inaccurate genetic linkage maps, all derived from relatively small F1 populations. Higher-density linkage maps, optical mapping, or other sources of long-range linkage information could offer more clues about the likely explanations. However, that such a small portion of contigs were problematic suggests that cacao breeding programs without linkage maps could scaffold their genome with publicly available linkage maps without a significant negative impact on the structural accuracy of the assembly. In the future, increasing the depth of MinION sequencing could conceivably replace the need for linkage maps or optical mapping if enough very long reads (> 500 kbps) are generated. Presumably, the cost of reagents will also decrease while throughput continues to increase.

Compared with each other, the minimap and canusm assemblies contained some notable differences. The minimap assembly had a lower contiguity and contained fewer misjoins. The smaller number of misjoins identified in the minimap assembly might be a direct consequence of having shorter contigs, which would span fewer loci on the linkage map. The minimap assembly had a higher degree of redundancy compared with the canusm. We were able to verify one case where redundant contigs did represent actual structural differentiation present in the Pound 7 genome; however, other instances could represent artifacts of the software pipeline used. Comparatively, the canusm assembly collapsed more haplotypes, and produced longer contigs; its higher contiguity leads to a higher percentage of the contigs anchored on the cacao linkage maps (Table 4). For the purposes of obtaining a representative reference genome for an otherwise uncharacterized species, the canusm method would seem to be the more sensible choice. For the purposes of studying a particular cultivar or individual, the minimap method could be the better choice, producing more information about the genomic content of that single lineage.

**Table 4:** Comparison of cacao genomes after scaffolding

	B97–61/B2	Matina 1–6	B97–61/B2	Pound 7 (minimap)		Pound 7 (canusm)	
Version/scaffolding	v1	v1.1	v2	With Pound 7 linkage map only	With published cacao linkage maps	With Pound 7 linkage map only	With published cacao linkage maps
Number of scaffolds	4792	714	431	1316	506	609	377
Assembly size (Mb)	326.9	346.7	324.9	379.3	379.4	344.6	344.7
Scaffold N50 (Mb)	0.5	34.4	36.4	1.8	37.5	18.7	36.9
Scaffold L50	178	5	5	16	5	7	5
Contig misjoins identified	–	–	–	0	3	4	9
Anchored contigs	–	–	–	212	593	170	402
Anchored Mb (% of assembly)	218.4 (66.8%)	330 (95.3%)	314.2 (96.7%)	178.5 (47.0%)	327.3 (86.3%)	210.6 (61.1%)	317.6 (92.2%)

### Inexpensive and accurate whole-genome sequences required for breeding and gene-editing applications could be generated in cacao in remote locations

For cacao and similar crops, the accurate sequencing of heterozygous haplotypes will be essential for accurate genome editing of the elite F_1_ hybrids that can increase cacao yields and the sustainability of the industry. Cacao yields are stagnant, and land use has expanded to meet increasing demand for chocolate (Fig. 1a, b). Modernizing breeding in remote and under-resourced locations where cacao is cultivated could speed yield gains on current farms, preventing predation of sensitive tropical ecosystems. The previous cacao genome projects required large scientific teams and took years to complete. Here, we rapidly sequenced a more complex clone, producing a similar quality genome for ~$5000 of supplies, with a small team, in a period of months. Ultimately, each breeding program could have high-quality reference genomes of their best, regionally adapted clones to guide breeding and genome editing.

When purchased in bulk, MinION flow cells can cost as little as $500, with $100 per library preparation. The assembly produced with five MinION flow cells had a contig N50 over 10 times larger than the B97–61/B2 v2 assembly, which contained Sanger BAC sequences combined with 52× coverage of PacBio long reads. Based on the BUSCO completeness score, the MinION-only assembly was of poor quality, but the addition of low-coverage Illumina short reads put it on par with the previous two cacao genomes (Table 2).

The MinION flow cells and reagents proved robust during the course of this project, despite several obstacles common in developing countries and remote locations: day-long power outages, seizure of flow cells by US Customs, and a hurricane evacuation. Even after nearly a week at room temperature at the US Customs facility in Memphis, the flow cell used in run number four (Table 1) performed at a relatively high level, generating nearly 3 gbps of throughput with a read N50 over 20 kbps. Our experiences suggest that the MinION and its reagents will allow this type of work to succeed in the remote locations where cacao is grown.

The bottleneck is however in the assembly process. Nevertheless, we believe that the sequencing pipeline could be packaged into a seamless, point-and-click interface. A complete toolkit for modernizing cacao or other tropical crops breeding programs could include DNA extraction buffers, large genomic DNA cleanup columns, a MinION, an inverter generator, and a laptop with the specialized software package pre-installed. This “portable core facility” would cost less than $10,000, and democratize genomics-enabled breeding. In addition to genetically improving these crops, this platform could help monitoring the evolution of human and crop pathogens, or genomics-based food safety tests. The qualities that make such a system portable also allow it to function as a centralized core facility in environments with limited infrastructure, like erratic electricity and poor delivery of scientific reagents. More resource-sensitive equipment (e.g., PacBio RSII) would likely not be sustainable in this type of environment.

The assemblies presented in this work are the first step toward demonstrating a more “personalized” approach to cacao genomics and lay the group work for further characterization of the genomic diversity present within *T. cacao*.

## Materials and methods

### Plant material and DNA preparation

The extraction of cacao DNA was simplified to better facilitate the transfer of this work to remote locations. Half of the mature leaf was collected from the Pound 7 (clone B) tree in the Mars Miami (Florida, USA) greenhouse. For the high-molecular-weight DNA used in four of the five flow cells, the leaf was ground in liquid nitrogen. For the fragmented library run on one of the five flow cells, the leaf was homogenized with metal beads in a tube shaker, rather than liquid nitrogen (the fragment size was confirmed by Tapestation immediately before loading onto the flow cell). Homogenized tissue was resuspended in 20 mL of modified CTAB buffer (2% cetyltrimethyl ammonium bromide, 1% polyvinylpolypyrrolidone (PVPP), 0.1 M Tris-HCl, pH 8.0, 0.02 M EDTA, 1.4 M NaCl, and 10% β-mercaptoethanol (added right before use)). This was incubated at 65 °C for 10 min. An equal volume of chloroform was added, and the mixture was shaken for 30 s to 1 min. The solution was spun at 3000 rpm for 30 min at 4 °C in a Sorvall centrifuge (or higher speed if performed in a microfuge). The supernatant was collected, and 0.7× volume of isopropanol was added. After mixing by gentle inversion, wispy DNA precipitated, was removed, and washed in 70% ethanol. The washed pellet was removed and put into a new tube and allowed to dry for 30 min, and then resuspended in 500 µL of nuclease-free water. If the 260/230 ratio was lower than 1.9 on a Nanodrop spectrometer, additional cleanup was performed using Qiagen G-tip 100 columns using the manufacturer’s protocol.

### 1D sequencing library construction and sequencing

Libraries were constructed using the Oxford Nanopore 1D kit, following the manufacturer’s protocol. The MinION sequencing was performed following the manufacturer’s protocol.

Basecalling of the raw sequencing was done with Albacore (v.2.0.2), which was run using the preset parameters for each kit and flowcell configuration (Table 1). Albacore’s default filtering was used to bin reads into “fail” and “pass” categories based on whether the mean phred-scaled quality score was lower or higher than 7.5, respectively.

A final, filtered set of reads was produced comprising those reads equal to or larger than 1000 bp and categorized as “pass” by Albacore.

### Linkage mapping

A F1 mapping population where Pound 7 was the mother has been previously described^5^. LepMap2^18^ was used to construct a maternal genetic linkage map from the SNP genotypes.

### Assembly

Two methods were used to assemble the filtered MinION reads into unpolished contigs. The first de novo assembly method used a pipeline based on the minimap, miniasm^29^, and racon^30^ with the parameters recommended for high-error-rate long reads. Recent papers have employed a similar protocol for MinION-based assemblies of yeast^22^ and Arabidopsis^23^. In short, the assembly followed this procedure: overlaps between reads were cataloged by the minimap, and then assembled into contigs by miniasm. These contigs were then iteratively refined four times, where the MinION reads were mapped to the current set of contigs with graphmap^31^, and then consensus correction was done with Racon to produce a new set of contigs to be used in the next iteration.

The second assembly followed a procedure based on canu^32^ and SMARTdenovo^33^, similar to the one presented in by Schmidt^16^. In short, the correction module of canu (v1.7) was first run on the raw MinION reads. SMARTdenovo was then used to assemble the corrected reads, and produce a set of consensus-corrected contigs.

Contigs representing the chloroplast and mitochondrial genomes were identified by comparing post-refinement contigs from the minimap assembly to the published cacao organelle genomes using nucmer^34^. The reads that mapped to these contigs were then reassembled with Circlator^25^. Coverages for each of these organelle genomes were much higher on average (~500× for mitochondrion and 1500× for chloroplast), which allowed for accurate correction with nanopolish^35^. The MinION reads from the chloroplast and mitochondrial genomes were aligned to the circularized assemblies with graphmap and then corrected with nanopolish using default settings.

The following steps were run separately on the contigs from the minimap and canu–smartdenovo assembly procedures:

### Polishing

A final set of polished contigs were created through five iterations of Pilon:^22^ the Illumina short reads were remapped to the contigs with BWA MEM^36^, duplicates were marked with picard, and then Pilon was run (–fix snps,indels) to produce a new set of contigs for the next round.

### Scaffolding

Long MinION reads (> = 20 kbp) and both sets of Illumina short reads were remapped to the Pilon-corrected contigs from the final iteration. Contig regions of 100 bp or more that had low coverage (1× or less) of both long MinION reads and proper short-read pairs were marked. The contigs were then split at both the beginning and ending positions of these marked regions.

A self-comparison of the split contigs was carried out with nucmer (v4.0.2beta) to identify a set of nonredundant contigs for scaffolding. Among pairs of contigs, haplotype redundancies and other highly repetitive regions were identified as those pairs where one contig overlapped the other at 70% or more of its positions at 90% or higher sequence identity, and where the alignment range on either contig was no greater than 2× the alignment range on the other. The shorter contig from each redundant pair was temporarily set aside from the assembly.

The nonredundant contigs were compared with six published SNP-based linkage maps with ALLMAPS^19^. Contigs that disagreed with the four or more of the linkage maps were split again, at each point of disagreement. These split contigs were then anchored to the linkage maps with ALLMAPS, where Pound 7-only map was assigned a weight of 10, and the others a weight of 1.

Contigs that had been previously split by low coverage, but those that were also anchored to adjacent map positions were rejoined to each other (i.e., concatenated after having removed or refilled the gap between them). The contigs both placed and unplaced by ALLMAPS were then combined with the redundant contigs to get a final draft set of contigs.

All assembly scripts, contig sets, and AGP files are available online here https://osf.io/kdtp6/?view_only=ec65067a081b4c2fbe29eec67ec65771.

### Validation

#### Genotypes

The SNP loci previously genotyped with an Illumina Infinium array^17^ were lifted over from their original positions on Matina v1.1 to the Pound 7 contigs using blat and other UCSC tools^37^. The alleles from the Pound 7 contigs were then checked for consistency against the Pound 7 genotypes called on the chip array.

#### BUSCO

BUSCO (v3) was used to check the completeness of the assembly at each step (with the embryophyta_odb9 gene set; ~1440 in total). A combination of blat, ALLMAPS^29^, and in-house R scripts^38^ were used to check that contigs were not being split within regions containing complete BUSCO hits.

#### Gene model comparisons

Gene models were predicted ab initio on the final draft contigs with Augustus v3.3.1^23^, parameterized with the “cacao” training set^5^. Transcript sequences were extracted from these predictions, and were also tested with BUSCO, in transcriptome mode (-m tran). Polished transcripts from the Criollo assembly were aligned to the “final draft” contigs of each Pound 7 assembly.

### Repeat masking

Repetitive elements were identified with RepeatMasker using the Viridiplantae library^39^ from the RepeatMasker Combined Database: Dfam_Consensus-20170127, RepBase-20170127, run with rmblastn version 2.2.27 +.

### BAC-based haplotype comparisons

Nucmer was used to identify Pound 7 contigs that shared significant overlap with two unpublished, BAC-based haplotype assemblies of an important QTL locus of Pound 7 (Supplementary Material). Gepard^40^ was used to visually compare Pound 7 contigs from the MinION assembly to both of the unpublished BAC-based haplotypes. These contigs were aligned to their corresponding BAC haplotype with Mauve, and these alignments were summarized using in-house R scripts based on the biostrings package.

### Data deposition

MinION, Illumina, and BAC sequences will be deposited in NCBI Bioproject PRJNA421343.

## Supplementary information

Supplemental Material
