# Supplementary material for: Low-cost assembly of a cacao crop genome is able to resolve complex heterozygous bubbles
Source: Hortic Res. 2019 Apr 6;6:44. doi: 10.1038/s41438-019-0125-7 (PMC6441652; doi:10.1038/s41438-019-0125-7)
Supplement: Supplementary file 1 — Supplemental Material [file 41438_2019_125_MOESM1_ESM.pptx]

## Slide 1
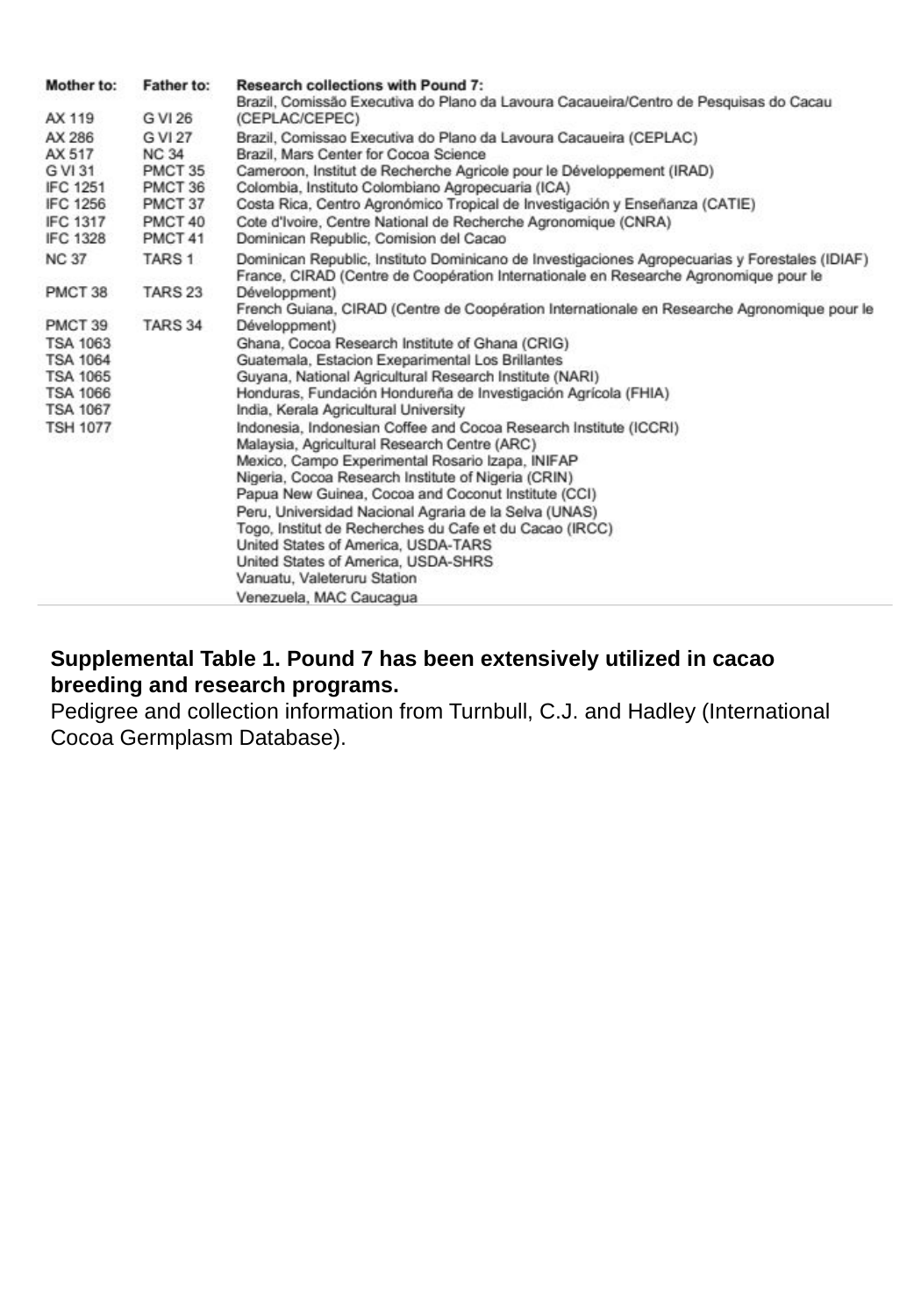

Supplemental Table 1. Pound 7 has been extensively utilized in cacao breeding and research programs.
Pedigree and collection information from Turnbull, C.J. and Hadley (International Cocoa Germplasm Database).

## Slide 2
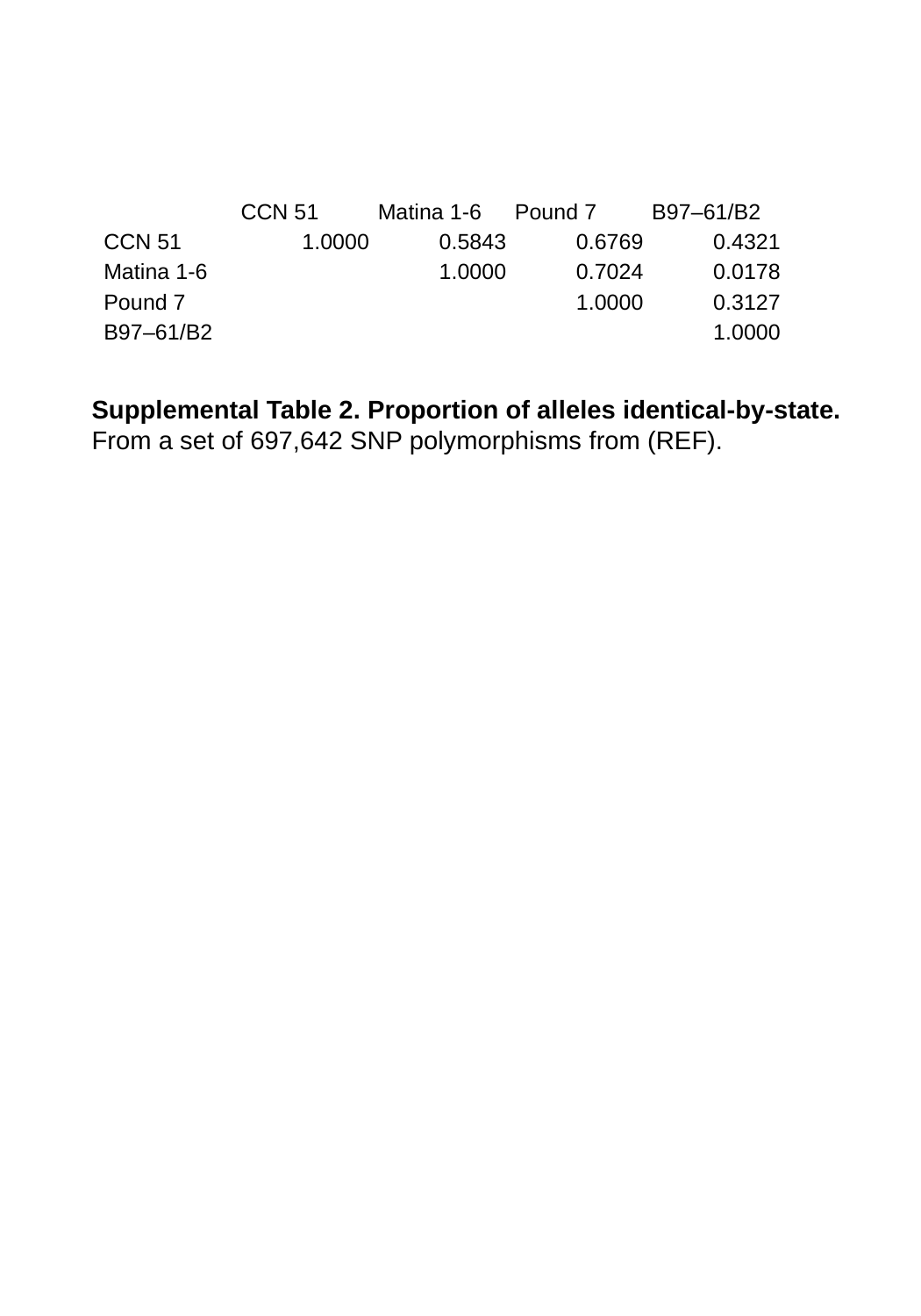

| | CCN 51 | Matina 1-6 | Pound 7 | B97–61/B2 |
| --- | --- | --- | --- | --- |
| CCN 51 | 1.0000 | 0.5843 | 0.6769 | 0.4321 |
| Matina 1-6 | | 1.0000 | 0.7024 | 0.0178 |
| Pound 7 | | | 1.0000 | 0.3127 |
| B97–61/B2 | | | | 1.0000 |
Supplemental Table 2. Proportion of alleles identical-by-state.
From a set of 697,642 SNP polymorphisms from (REF).

## Slide 3
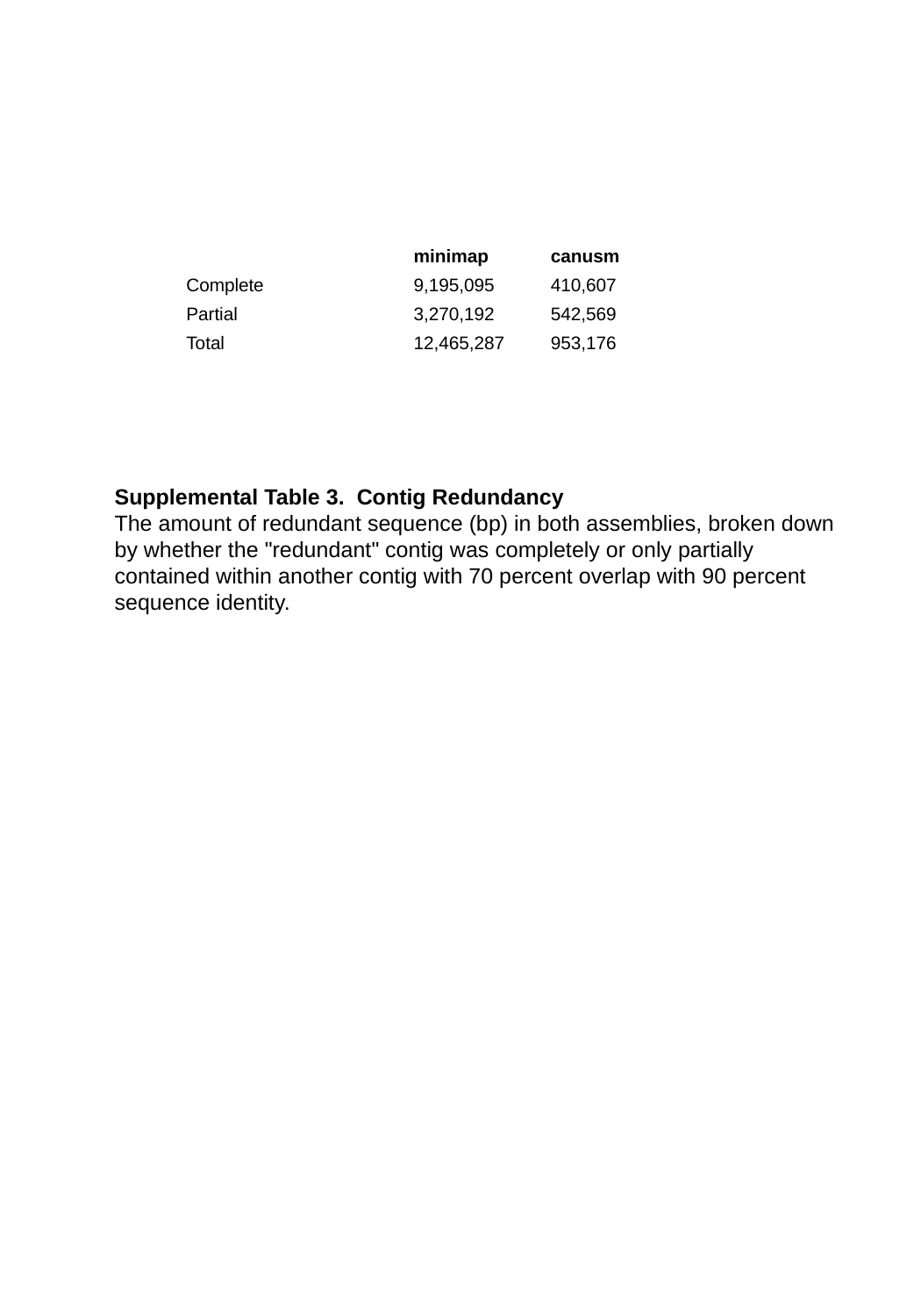

| | minimap | canusm |
| --- | --- | --- |
| Complete | 9,195,095 | 410,607 |
| Partial | 3,270,192 | 542,569 |
| Total | 12,465,287 | 953,176 |
Supplemental Table 3. Contig Redundancy
The amount of redundant sequence (bp) in both assemblies, broken down by whether the "redundant" contig was completely or only partially contained within another contig with 70 percent overlap with 90 percent sequence identity.

## Slide 4
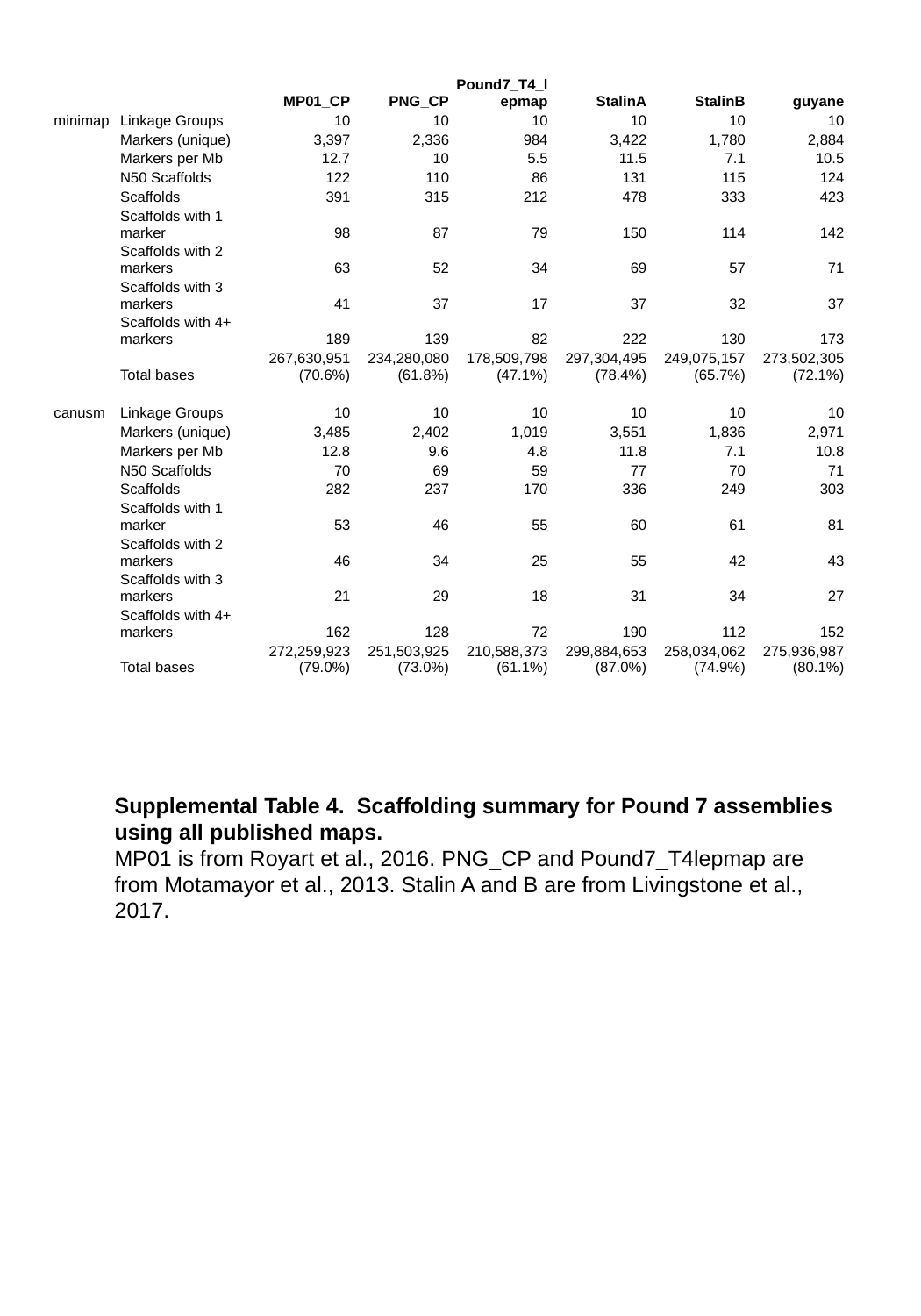

| | | MP01\_CP | PNG\_CP | Pound7\_T4\_lepmap | StalinA | StalinB | guyane |
| --- | --- | --- | --- | --- | --- | --- | --- |
| minimap | Linkage Groups | 10 | 10 | 10 | 10 | 10 | 10 |
| | Markers (unique) | 3,397 | 2,336 | 984 | 3,422 | 1,780 | 2,884 |
| | Markers per Mb | 12.7 | 10 | 5.5 | 11.5 | 7.1 | 10.5 |
| | N50 Scaffolds | 122 | 110 | 86 | 131 | 115 | 124 |
| | Scaffolds | 391 | 315 | 212 | 478 | 333 | 423 |
| | Scaffolds with 1 marker | 98 | 87 | 79 | 150 | 114 | 142 |
| | Scaffolds with 2 markers | 63 | 52 | 34 | 69 | 57 | 71 |
| | Scaffolds with 3 markers | 41 | 37 | 17 | 37 | 32 | 37 |
| | Scaffolds with 4+ markers | 189 | 139 | 82 | 222 | 130 | 173 |
| | Total bases | 267,630,951 (70.6%) | 234,280,080 (61.8%) | 178,509,798 (47.1%) | 297,304,495 (78.4%) | 249,075,157 (65.7%) | 273,502,305 (72.1%) |
| | | | | | | | |
| canusm | Linkage Groups | 10 | 10 | 10 | 10 | 10 | 10 |
| | Markers (unique) | 3,485 | 2,402 | 1,019 | 3,551 | 1,836 | 2,971 |
| | Markers per Mb | 12.8 | 9.6 | 4.8 | 11.8 | 7.1 | 10.8 |
| | N50 Scaffolds | 70 | 69 | 59 | 77 | 70 | 71 |
| | Scaffolds | 282 | 237 | 170 | 336 | 249 | 303 |
| | Scaffolds with 1 marker | 53 | 46 | 55 | 60 | 61 | 81 |
| | Scaffolds with 2 markers | 46 | 34 | 25 | 55 | 42 | 43 |
| | Scaffolds with 3 markers | 21 | 29 | 18 | 31 | 34 | 27 |
| | Scaffolds with 4+ markers | 162 | 128 | 72 | 190 | 112 | 152 |
| | Total bases | 272,259,923 (79.0%) | 251,503,925 (73.0%) | 210,588,373 (61.1%) | 299,884,653 (87.0%) | 258,034,062 (74.9%) | 275,936,987 (80.1%) |
Supplemental Table 4. Scaffolding summary for Pound 7 assemblies using all published maps.
MP01 is from Royart et al., 2016. PNG_CP and Pound7_T4lepmap are from Motamayor et al., 2013. Stalin A and B are from Livingstone et al., 2017.

## Slide 5
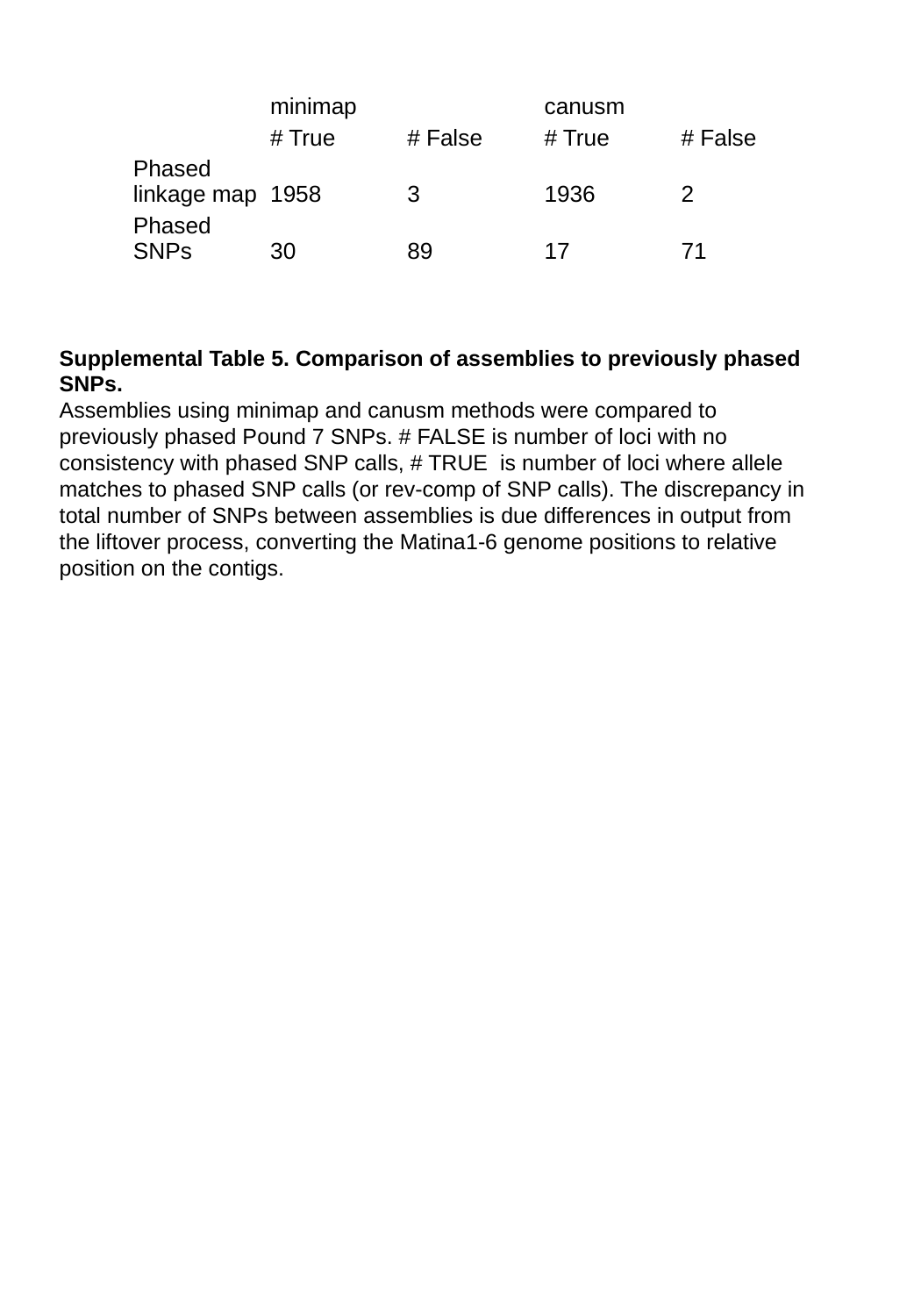

| | minimap | | canusm | |
| --- | --- | --- | --- | --- |
| | # True | # False | # True | # False |
| Phased linkage map | 1958 | 3 | 1936 | 2 |
| Phased SNPs | 30 | 89 | 17 | 71 |
Supplemental Table 5. Comparison of assemblies to previously phased SNPs.
Assemblies using minimap and canusm methods were compared to previously phased Pound 7 SNPs. # FALSE is number of loci with no consistency with phased SNP calls, # TRUE is number of loci where allele matches to phased SNP calls (or rev-comp of SNP calls). The discrepancy in total number of SNPs between assemblies is due differences in output from the liftover process, converting the Matina1-6 genome positions to relative position on the contigs.

## Slide 6
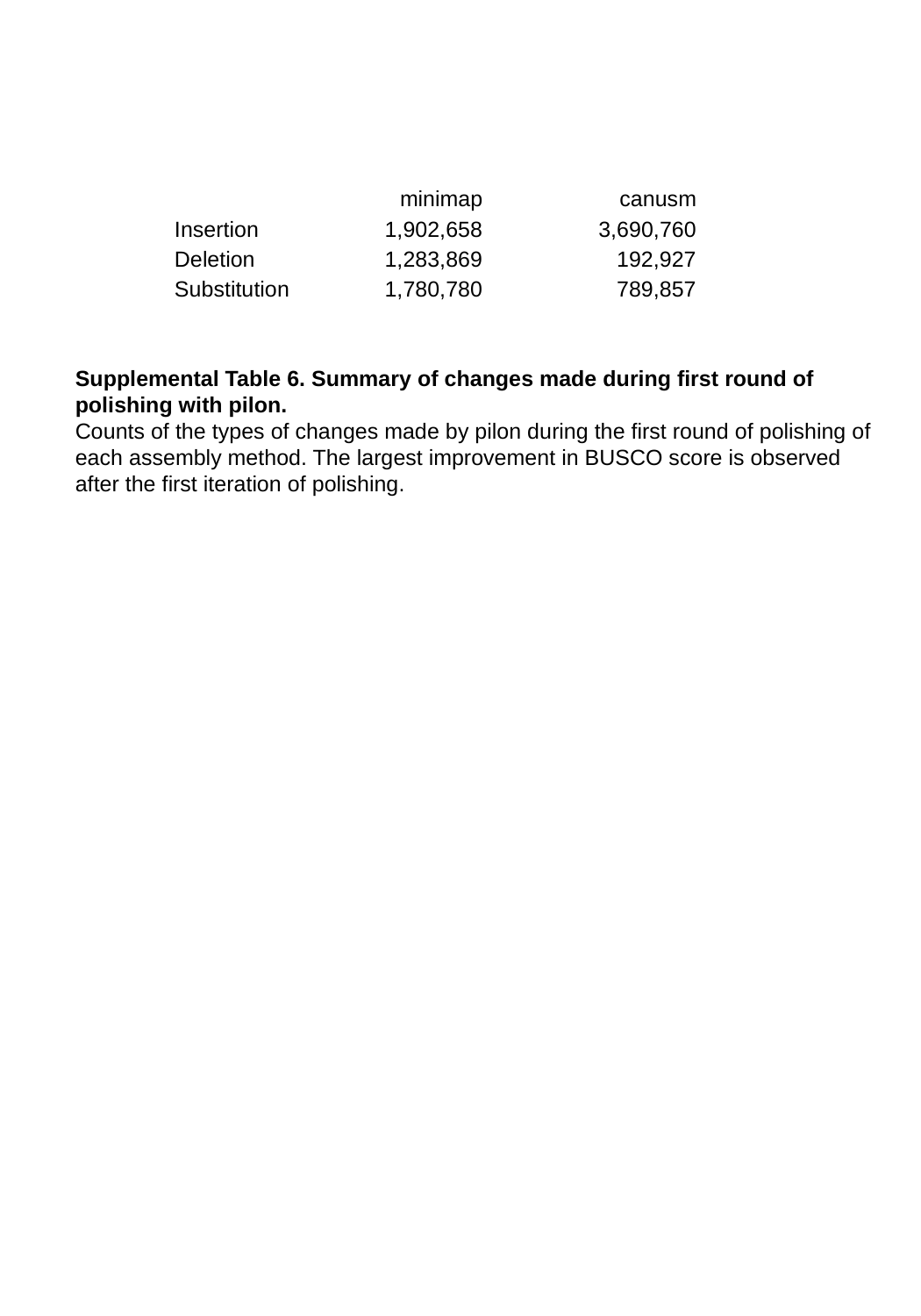

| | minimap | canusm |
| --- | --- | --- |
| Insertion | 1,902,658 | 3,690,760 |
| Deletion | 1,283,869 | 192,927 |
| Substitution | 1,780,780 | 789,857 |
Supplemental Table 6. Summary of changes made during first round of polishing with pilon.
Counts of the types of changes made by pilon during the first round of polishing of each assembly method. The largest improvement in BUSCO score is observed after the first iteration of polishing.

## Slide 7
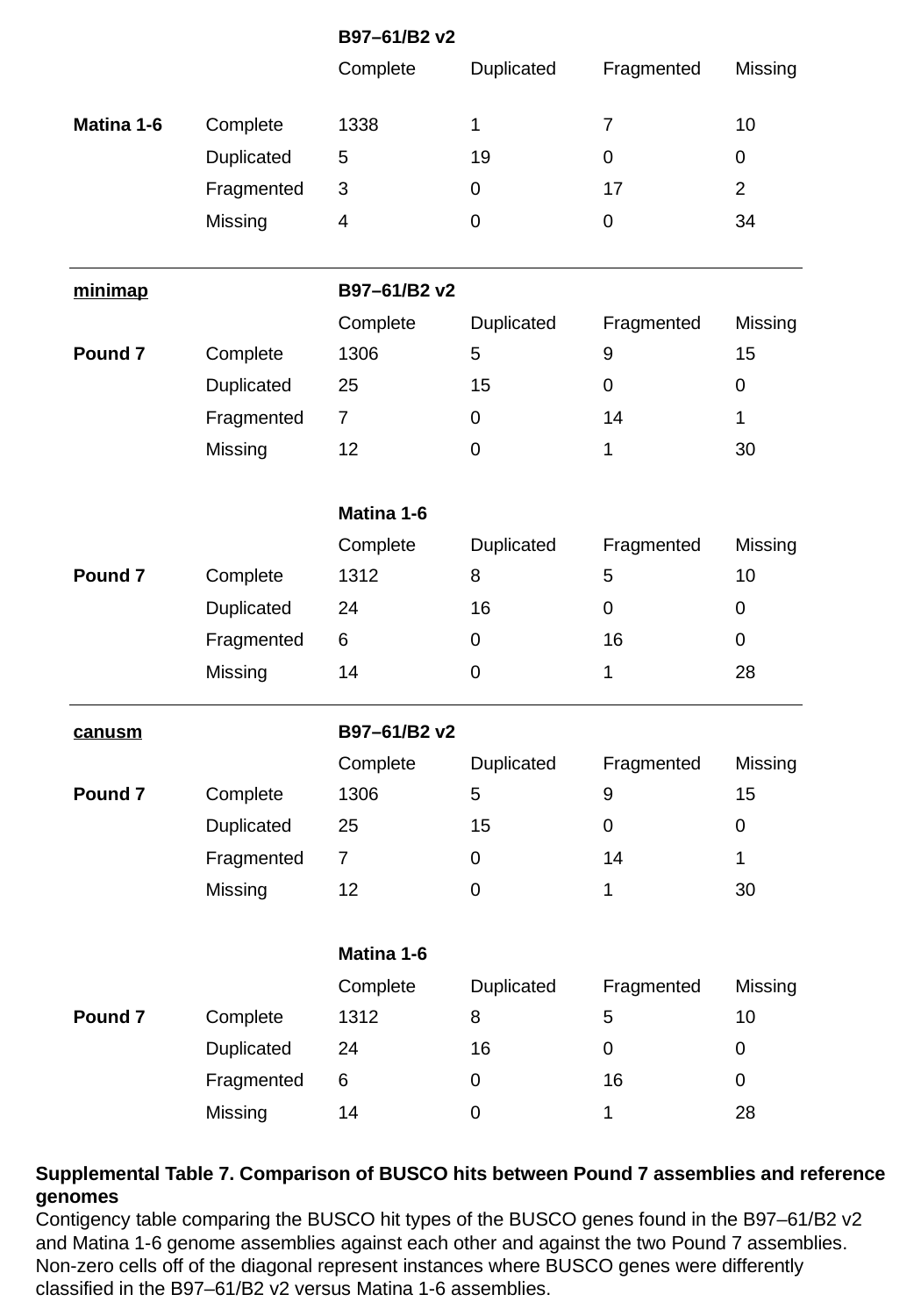

| | | B97–61/B2 v2 | | | |
| --- | --- | --- | --- | --- | --- |
| | | Complete | Duplicated | Fragmented | Missing |
| Matina 1-6 | Complete | 1338 | 1 | 7 | 10 |
| | Duplicated | 5 | 19 | 0 | 0 |
| | Fragmented | 3 | 0 | 17 | 2 |
| | Missing | 4 | 0 | 0 | 34 |
| minimap | | B97–61/B2 v2 | | | |
| --- | --- | --- | --- | --- | --- |
| | | Complete | Duplicated | Fragmented | Missing |
| Pound 7 | Complete | 1306 | 5 | 9 | 15 |
| | Duplicated | 25 | 15 | 0 | 0 |
| | Fragmented | 7 | 0 | 14 | 1 |
| | Missing | 12 | 0 | 1 | 30 |
| | | | | | |
| | | Matina 1-6 | | | |
| | | Complete | Duplicated | Fragmented | Missing |
| Pound 7 | Complete | 1312 | 8 | 5 | 10 |
| | Duplicated | 24 | 16 | 0 | 0 |
| | Fragmented | 6 | 0 | 16 | 0 |
| | Missing | 14 | 0 | 1 | 28 |
| canusm | | B97–61/B2 v2 | | | |
| --- | --- | --- | --- | --- | --- |
| | | Complete | Duplicated | Fragmented | Missing |
| Pound 7 | Complete | 1306 | 5 | 9 | 15 |
| | Duplicated | 25 | 15 | 0 | 0 |
| | Fragmented | 7 | 0 | 14 | 1 |
| | Missing | 12 | 0 | 1 | 30 |
| | | | | | |
| | | Matina 1-6 | | | |
| | | Complete | Duplicated | Fragmented | Missing |
| Pound 7 | Complete | 1312 | 8 | 5 | 10 |
| | Duplicated | 24 | 16 | 0 | 0 |
| | Fragmented | 6 | 0 | 16 | 0 |
| | Missing | 14 | 0 | 1 | 28 |
Supplemental Table 7. Comparison of BUSCO hits between Pound 7 assemblies and reference genomes
Contigency table comparing the BUSCO hit types of the BUSCO genes found in the B97–61/B2 v2 and Matina 1-6 genome assemblies against each other and against the two Pound 7 assemblies. Non-zero cells off of the diagonal represent instances where BUSCO genes were differently classified in the B97–61/B2 v2 versus Matina 1-6 assemblies.

## Slide 8
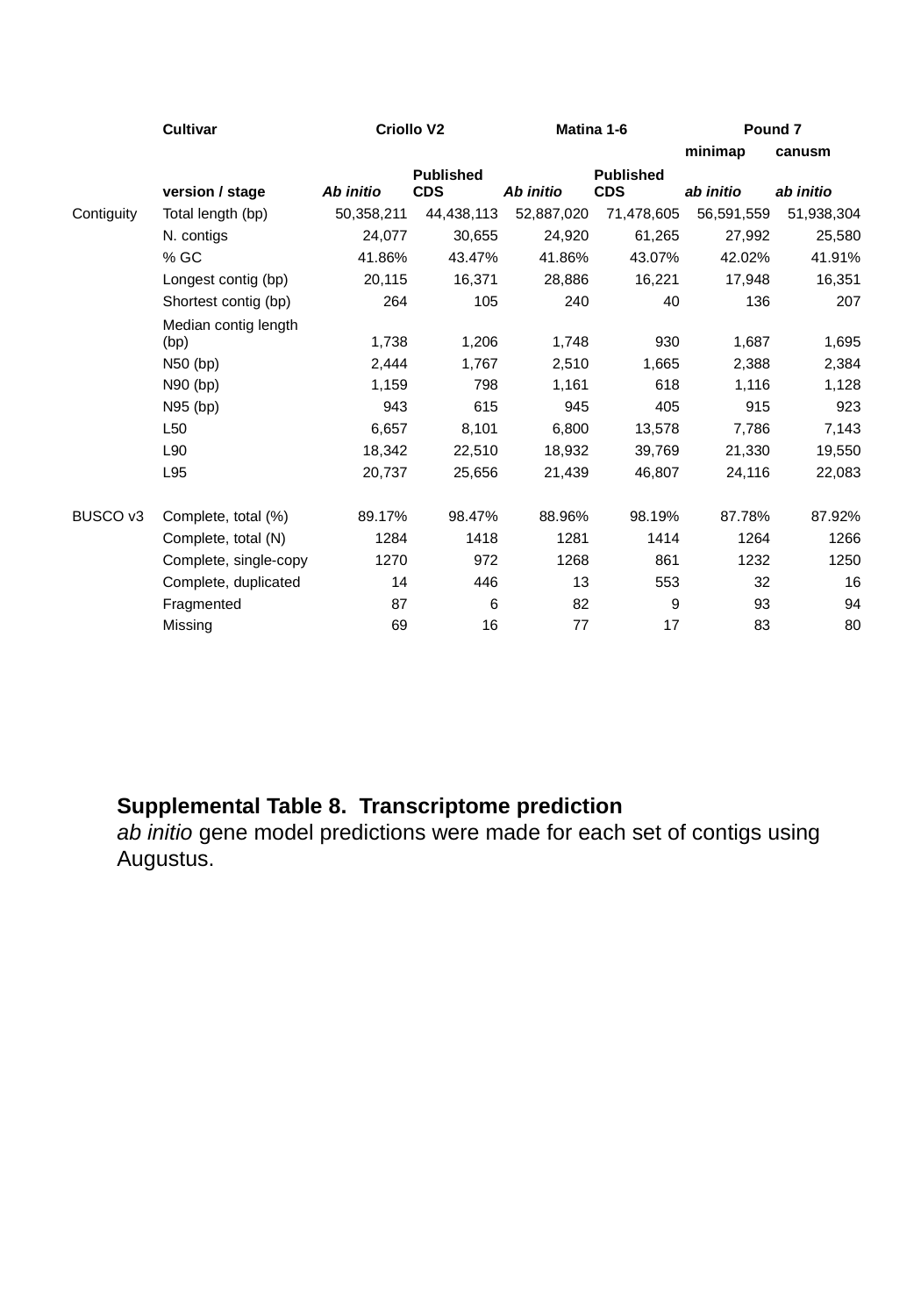

| | Cultivar | Criollo V2 | | Matina 1-6 | | Pound 7 | |
| --- | --- | --- | --- | --- | --- | --- | --- |
| | | | | | | minimap | canusm |
| | version / stage | Ab initio | Published CDS | Ab initio | Published CDS | ab initio | ab initio |
| Contiguity | Total length (bp) | 50,358,211 | 44,438,113 | 52,887,020 | 71,478,605 | 56,591,559 | 51,938,304 |
| | N. contigs | 24,077 | 30,655 | 24,920 | 61,265 | 27,992 | 25,580 |
| | % GC | 41.86% | 43.47% | 41.86% | 43.07% | 42.02% | 41.91% |
| | Longest contig (bp) | 20,115 | 16,371 | 28,886 | 16,221 | 17,948 | 16,351 |
| | Shortest contig (bp) | 264 | 105 | 240 | 40 | 136 | 207 |
| | Median contig length (bp) | 1,738 | 1,206 | 1,748 | 930 | 1,687 | 1,695 |
| | N50 (bp) | 2,444 | 1,767 | 2,510 | 1,665 | 2,388 | 2,384 |
| | N90 (bp) | 1,159 | 798 | 1,161 | 618 | 1,116 | 1,128 |
| | N95 (bp) | 943 | 615 | 945 | 405 | 915 | 923 |
| | L50 | 6,657 | 8,101 | 6,800 | 13,578 | 7,786 | 7,143 |
| | L90 | 18,342 | 22,510 | 18,932 | 39,769 | 21,330 | 19,550 |
| | L95 | 20,737 | 25,656 | 21,439 | 46,807 | 24,116 | 22,083 |
| | | | | | | | |
| BUSCO v3 | Complete, total (%) | 89.17% | 98.47% | 88.96% | 98.19% | 87.78% | 87.92% |
| | Complete, total (N) | 1284 | 1418 | 1281 | 1414 | 1264 | 1266 |
| | Complete, single-copy | 1270 | 972 | 1268 | 861 | 1232 | 1250 |
| | Complete, duplicated | 14 | 446 | 13 | 553 | 32 | 16 |
| | Fragmented | 87 | 6 | 82 | 9 | 93 | 94 |
| | Missing | 69 | 16 | 77 | 17 | 83 | 80 |
Supplemental Table 8. Transcriptome prediction
ab initio gene model predictions were made for each set of contigs using Augustus.

## Slide 9
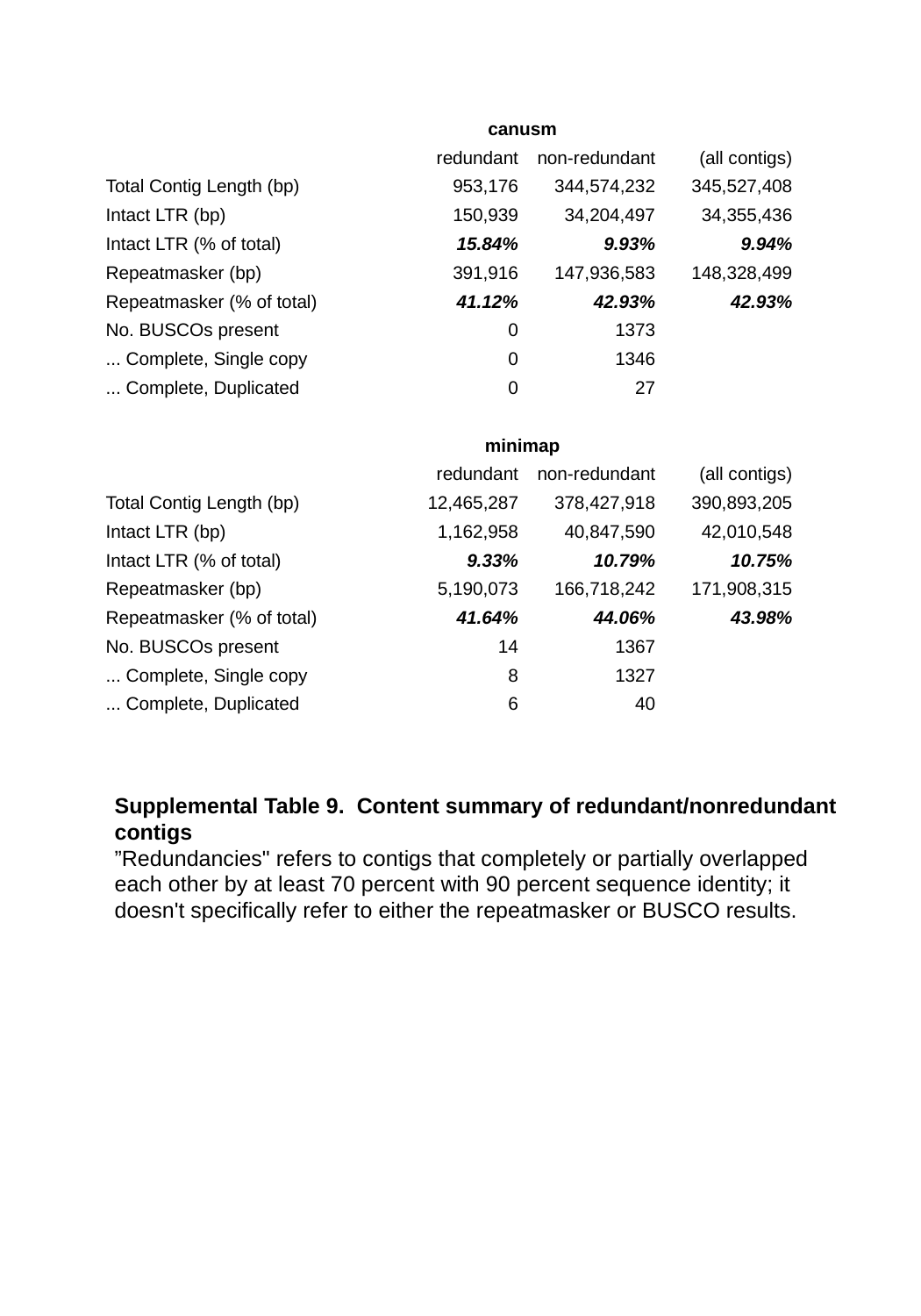

| | canusm | | |
| --- | --- | --- | --- |
| | redundant | non-redundant | (all contigs) |
| Total Contig Length (bp) | 953,176 | 344,574,232 | 345,527,408 |
| Intact LTR (bp) | 150,939 | 34,204,497 | 34,355,436 |
| Intact LTR (% of total) | 15.84% | 9.93% | 9.94% |
| Repeatmasker (bp) | 391,916 | 147,936,583 | 148,328,499 |
| Repeatmasker (% of total) | 41.12% | 42.93% | 42.93% |
| No. BUSCOs present | 0 | 1373 | |
| ... Complete, Single copy | 0 | 1346 | |
| ... Complete, Duplicated | 0 | 27 | |
| | | | |
| | minimap | | |
| | redundant | non-redundant | (all contigs) |
| Total Contig Length (bp) | 12,465,287 | 378,427,918 | 390,893,205 |
| Intact LTR (bp) | 1,162,958 | 40,847,590 | 42,010,548 |
| Intact LTR (% of total) | 9.33% | 10.79% | 10.75% |
| Repeatmasker (bp) | 5,190,073 | 166,718,242 | 171,908,315 |
| Repeatmasker (% of total) | 41.64% | 44.06% | 43.98% |
| No. BUSCOs present | 14 | 1367 | |
| ... Complete, Single copy | 8 | 1327 | |
| ... Complete, Duplicated | 6 | 40 | |
Supplemental Table 9. Content summary of redundant/nonredundant contigs
”Redundancies" refers to contigs that completely or partially overlapped each other by at least 70 percent with 90 percent sequence identity; it doesn't specifically refer to either the repeatmasker or BUSCO results.

## Slide 10
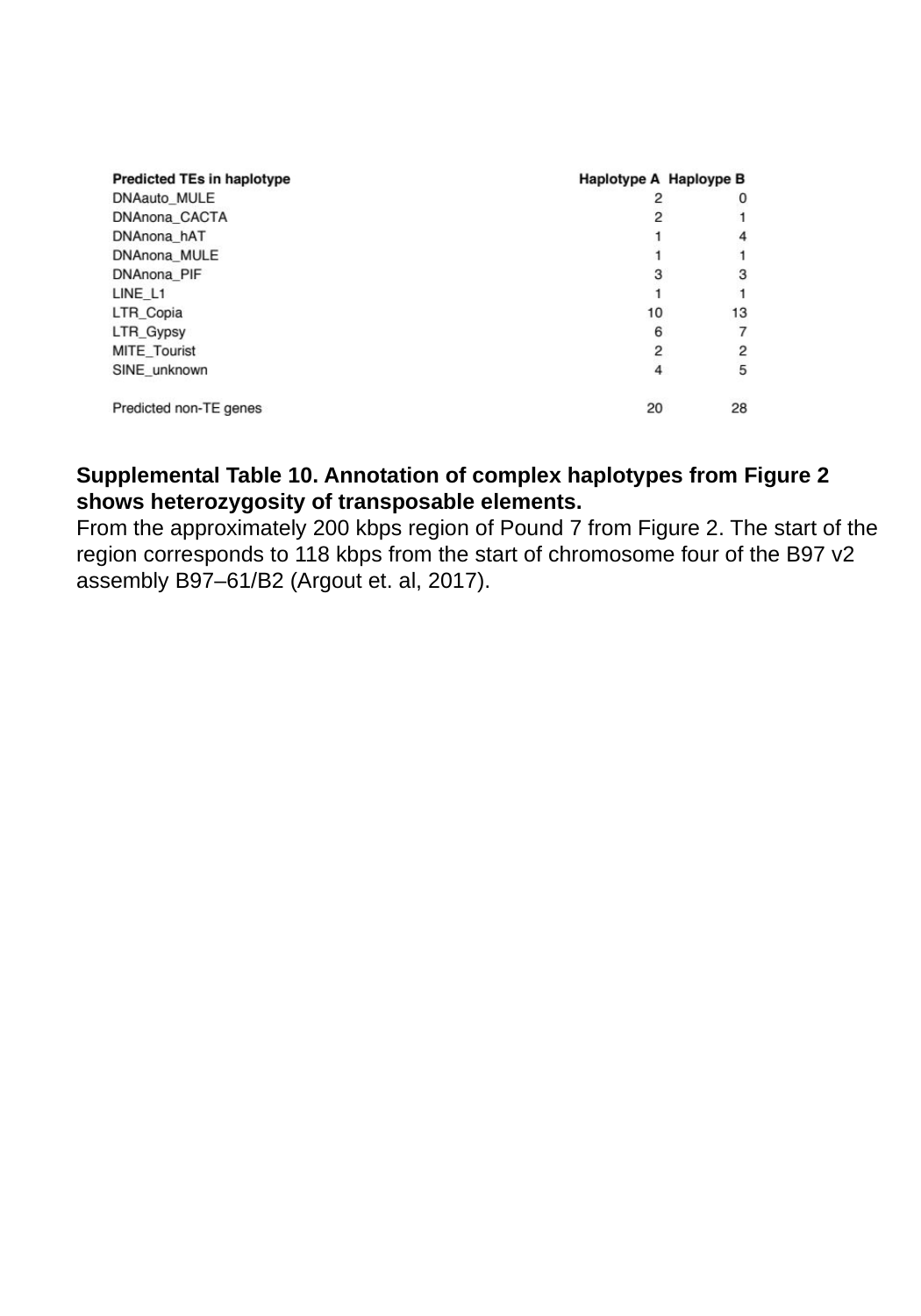

Supplemental Table 10. Annotation of complex haplotypes from Figure 2 shows heterozygosity of transposable elements.
From the approximately 200 kbps region of Pound 7 from Figure 2. The start of the region corresponds to 118 kbps from the start of chromosome four of the B97 v2 assembly B97–61/B2 (Argout et. al, 2017).

## Slide 11
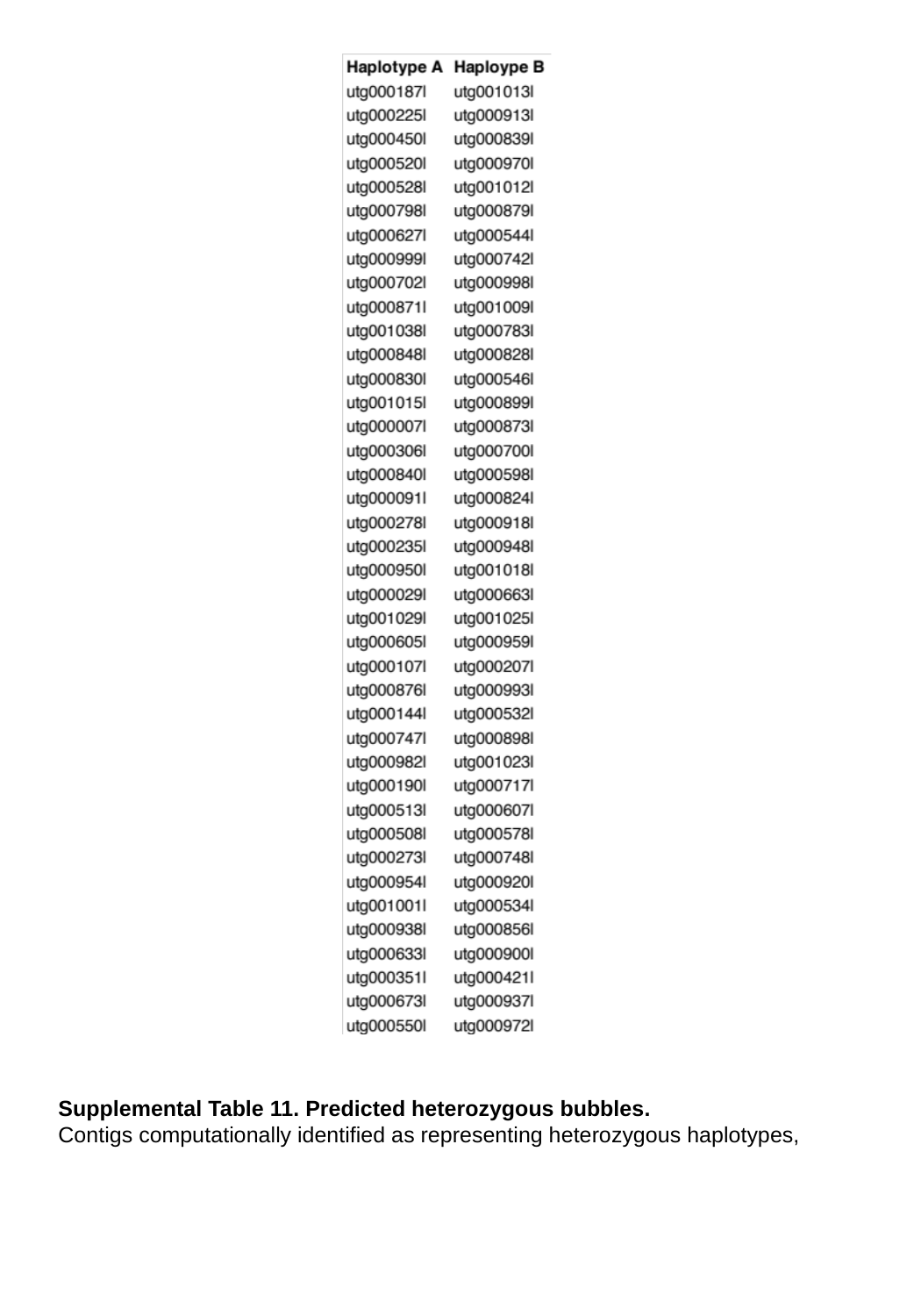

Supplemental Table 11. Predicted heterozygous bubbles.
Contigs computationally identified as representing heterozygous haplotypes,

## Slide 12
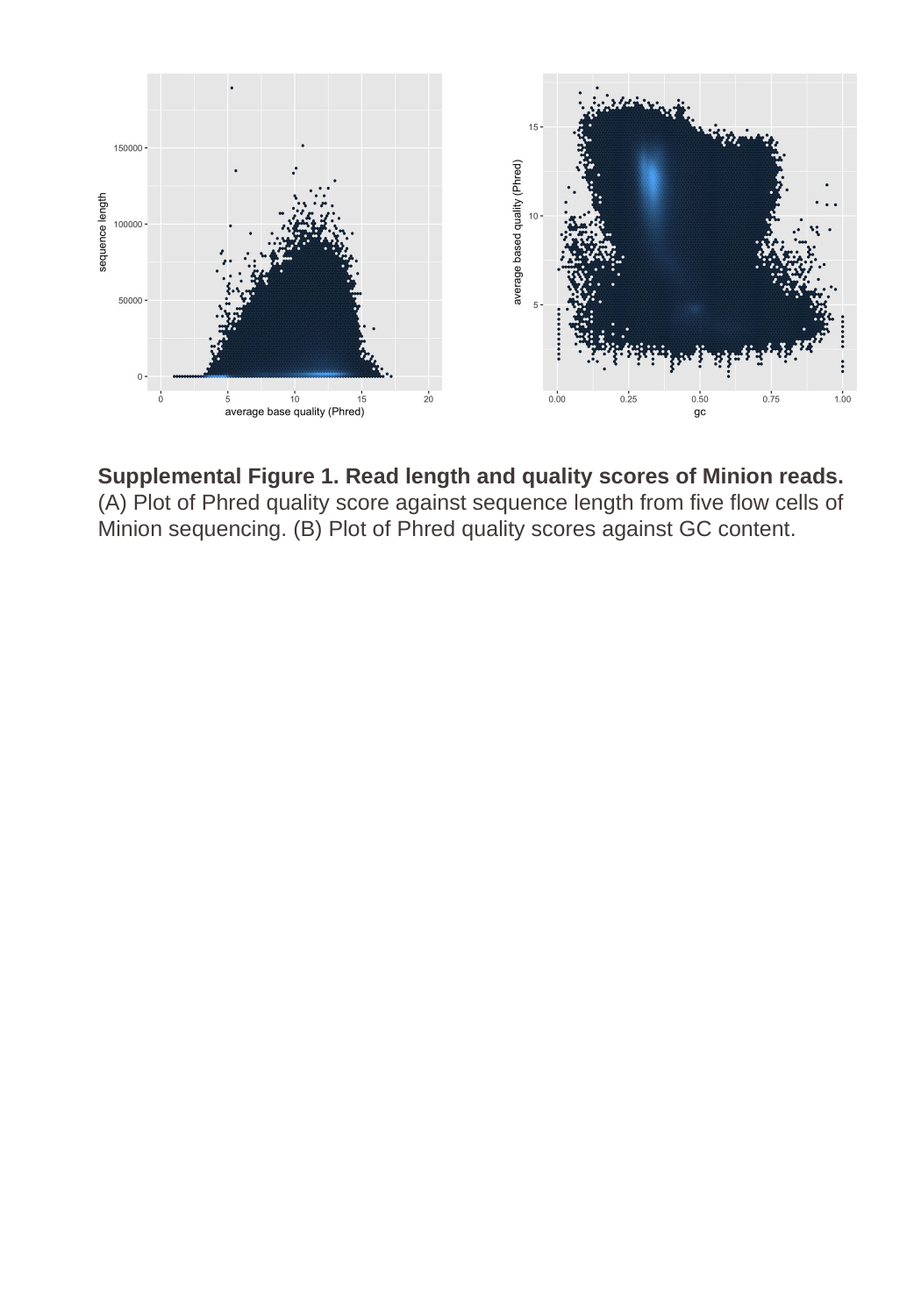

Supplemental Figure 1. Read length and quality scores of Minion reads.
(A) Plot of Phred quality score against sequence length from five flow cells of Minion sequencing. (B) Plot of Phred quality scores against GC content.

## Slide 13
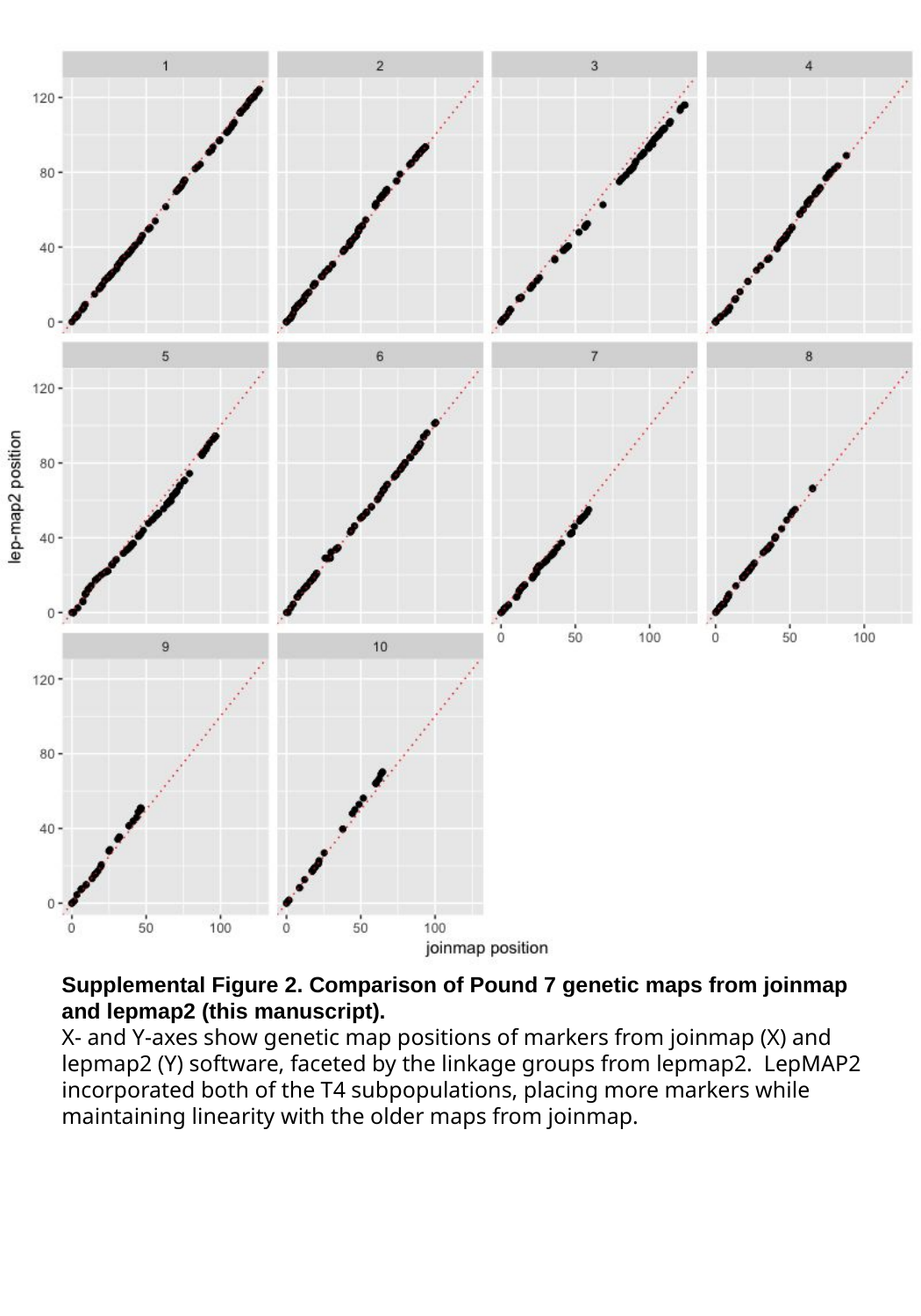

Supplemental Figure 2. Comparison of Pound 7 genetic maps from joinmap and lepmap2 (this manuscript).
X- and Y-axes show genetic map positions of markers from joinmap (X) and lepmap2 (Y) software, faceted by the linkage groups from lepmap2.  LepMAP2 incorporated both of the T4 subpopulations, placing more markers while maintaining linearity with the older maps from joinmap.

## Slide 14
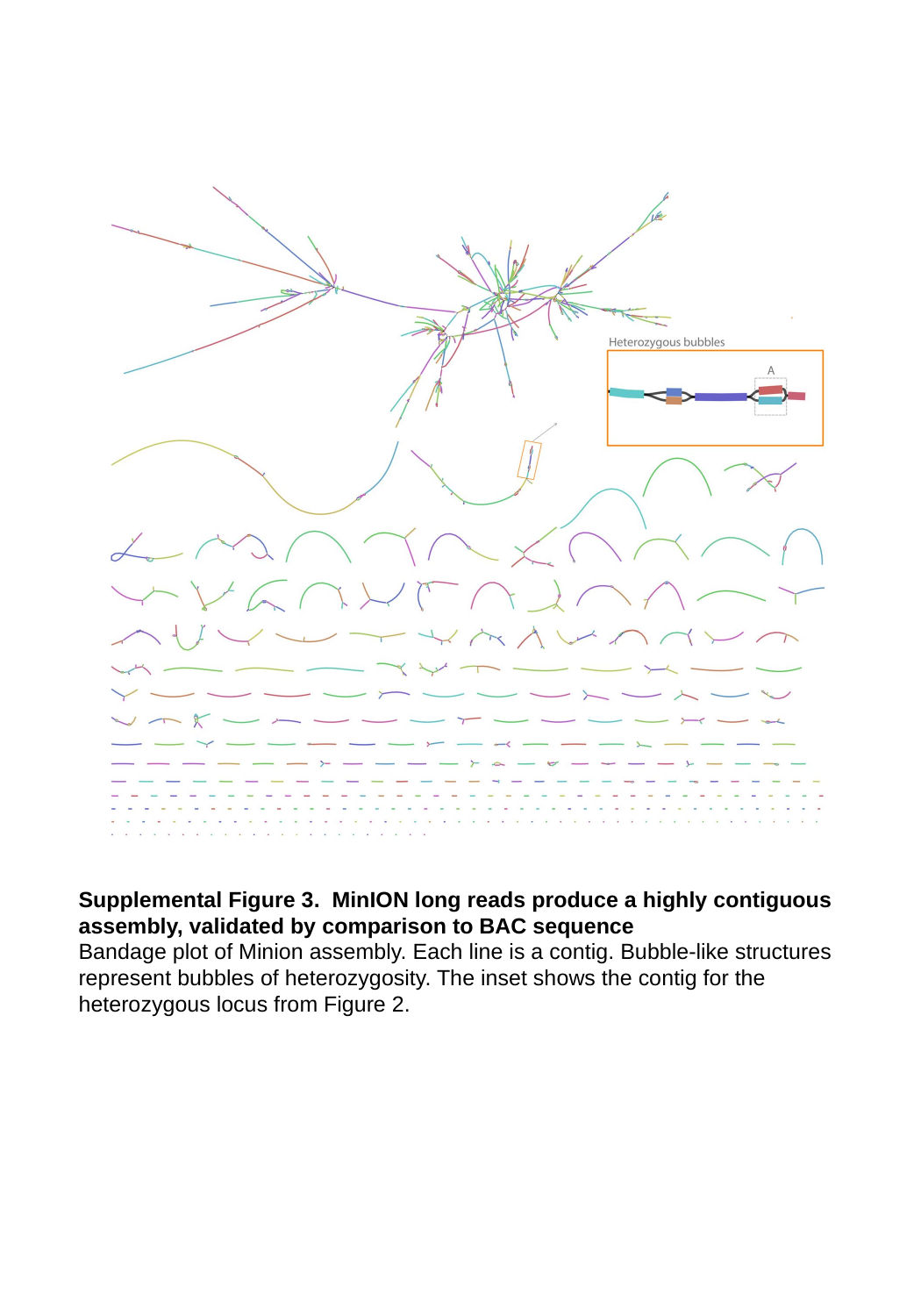

Supplemental Figure 3.  MinION long reads produce a highly contiguous assembly, validated by comparison to BAC sequence
Bandage plot of Minion assembly. Each line is a contig. Bubble-like structures represent bubbles of heterozygosity. The inset shows the contig for the heterozygous locus from Figure 2.

## Slide 15
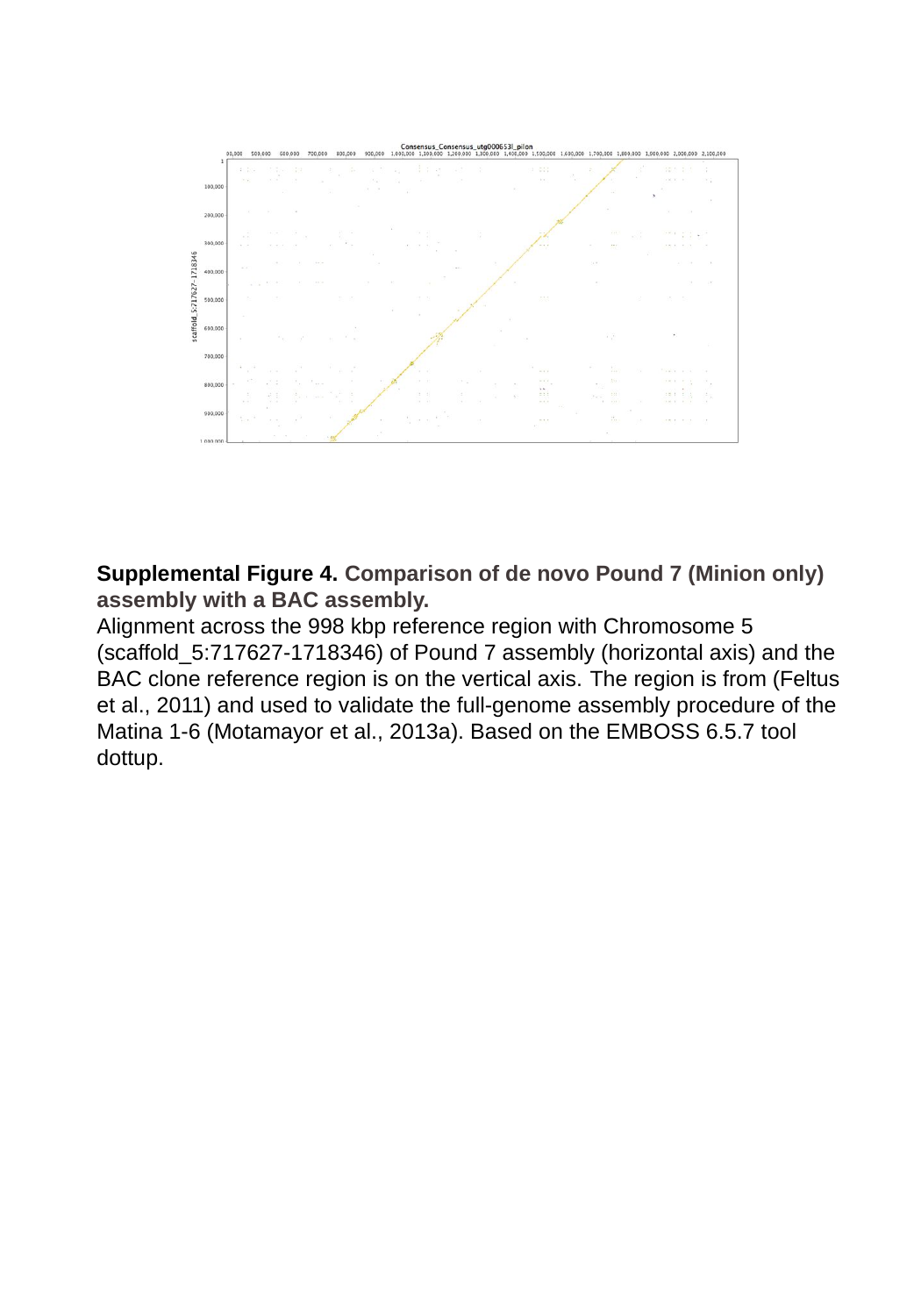

Supplemental Figure 4. Comparison of de novo Pound 7 (Minion only) assembly with a BAC assembly.
Alignment across the 998 kbp reference region with Chromosome 5 (scaffold_5:717627-1718346) of Pound 7 assembly (horizontal axis) and the BAC clone reference region is on the vertical axis. The region is from (Feltus et al., 2011) and used to validate the full-genome assembly procedure of the Matina 1-6 (Motamayor et al., 2013a). Based on the EMBOSS 6.5.7 tool dottup.

## Slide 16
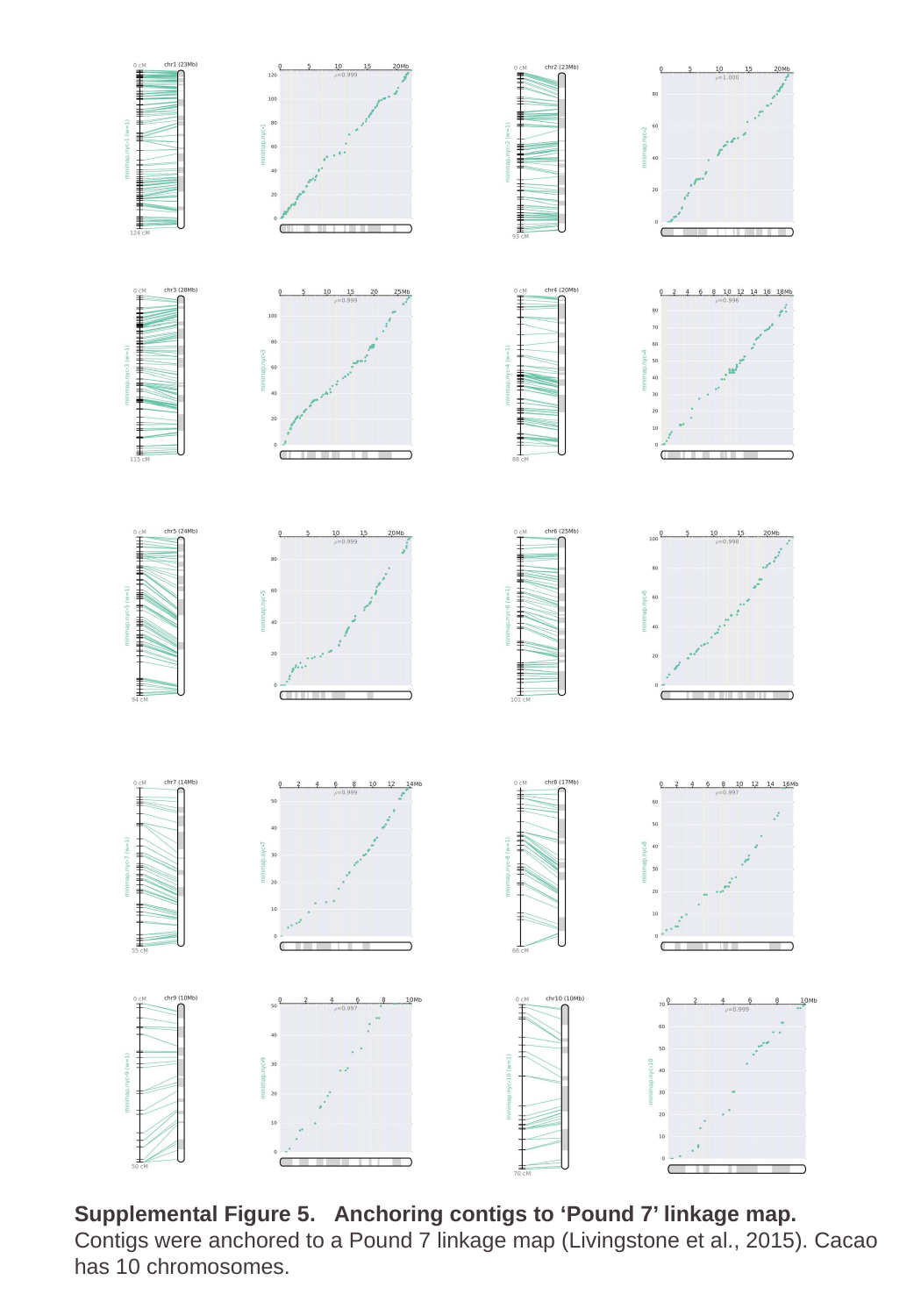

Supplemental Figure 5.  Anchoring contigs to ‘Pound 7’ linkage map.
Contigs were anchored to a Pound 7 linkage map (Livingstone et al., 2015). Cacao has 10 chromosomes.

## Slide 17
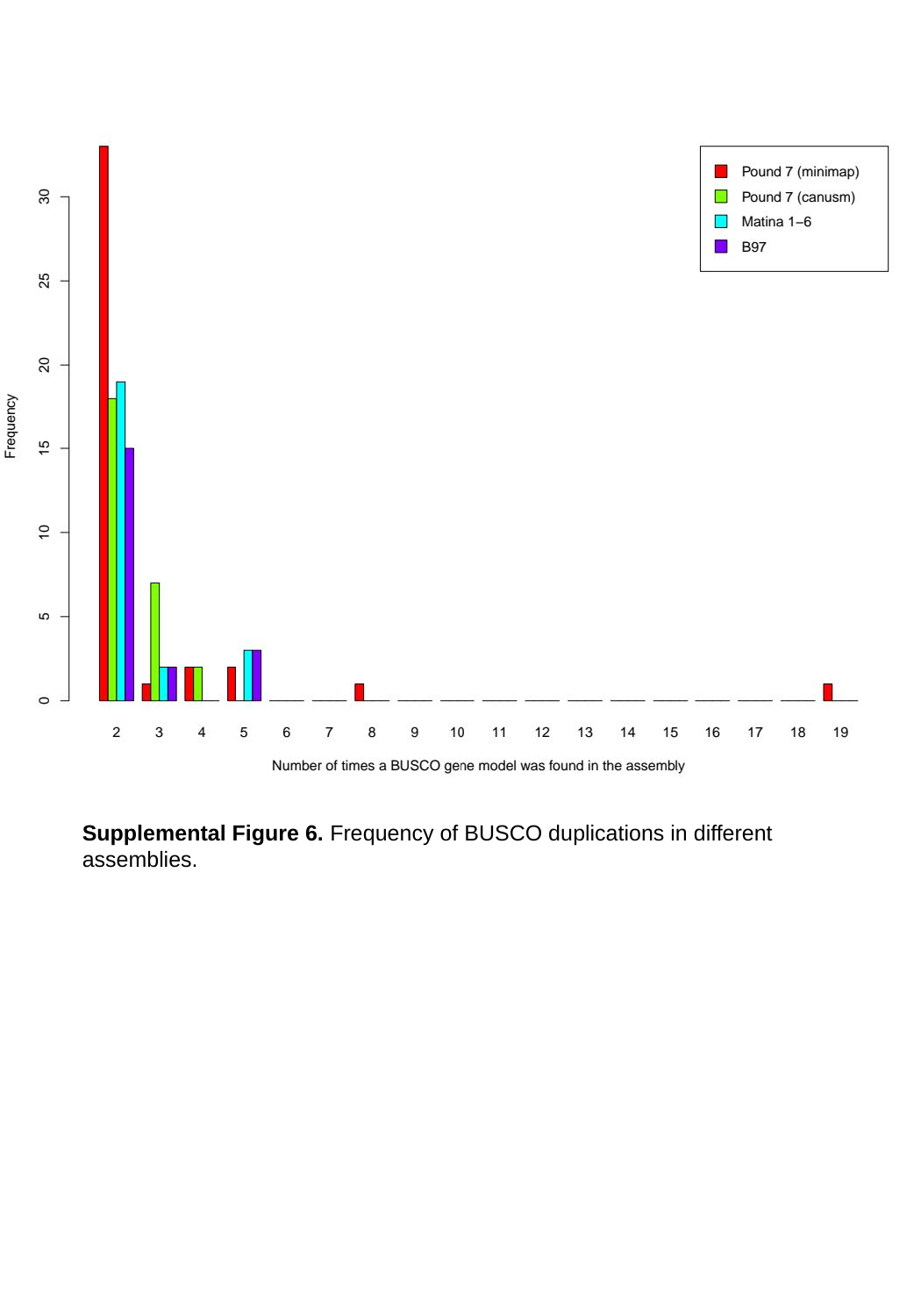

Supplemental Figure 6. Frequency of BUSCO duplications in different assemblies.

## Slide 18
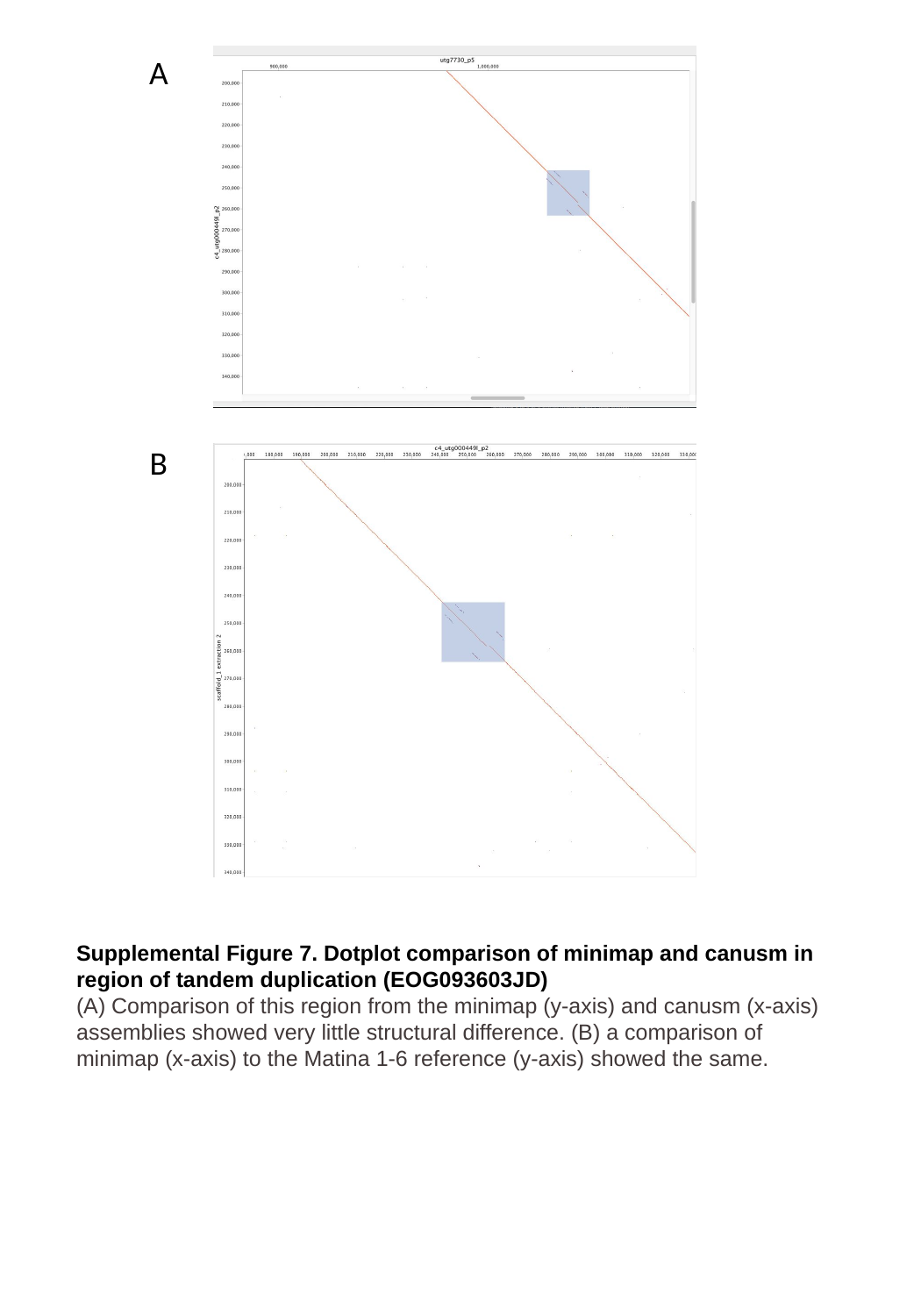

A
B
Supplemental Figure 7. Dotplot comparison of minimap and canusm in region of tandem duplication (EOG093603JD)
(A) Comparison of this region from the minimap (y-axis) and canusm (x-axis) assemblies showed very little structural difference. (B) a comparison of minimap (x-axis) to the Matina 1-6 reference (y-axis) showed the same.
